# Diversification of the type IV filament superfamily into machines for adhesion, protein secretion, DNA uptake, and motility

**DOI:** 10.1371/journal.pbio.3000390

**Published:** 2019-07-19

**Authors:** Rémi Denise, Sophie S. Abby, Eduardo P. C. Rocha

**Affiliations:** 1 Microbial Evolutionary Genomics, Institut Pasteur, CNRS, UMR3525, Paris, France; 2 Sorbonne Université, Collège doctoral, Paris, France; 3 Université Grenoble Alpes, CNRS, Grenoble INP, TIMC-IMAG, Grenoble, France; Imperial College London, UNITED KINGDOM

## Abstract

Processes of molecular innovation require tinkering and shifting in the function of existing genes. How this occurs in terms of molecular evolution at long evolutionary scales remains poorly understood. Here, we analyse the natural history of a vast group of membrane-associated molecular systems in Bacteria and Archaea—the type IV filament (TFF) superfamily—that diversified in systems involved in flagellar or twitching motility, adhesion, protein secretion, and DNA uptake. The phylogeny of the thousands of detected systems suggests they may have been present in the last universal common ancestor. From there, two lineages—a bacterial and an archaeal—diversified by multiple gene duplications, gene fissions and deletions, and accretion of novel components. Surprisingly, we find that the ‘tight adherence’ (Tad) systems originated from the interkingdom transfer from Archaea to Bacteria of a system resembling the ‘EppA-dependent’ (Epd) pilus and were associated with the acquisition of a secretin. The phylogeny and content of ancestral systems suggest that initial bacterial pili were engaged in cell motility and/or DNA uptake. In contrast, specialised protein secretion systems arose several times independently and much later in natural history. The functional diversification of the TFF superfamily was accompanied by genetic rearrangements with implications for genetic regulation and horizontal gene transfer: systems encoded in fewer loci were more frequently exchanged between taxa. This may have contributed to their rapid evolution and spread across Bacteria and Archaea. Hence, the evolutionary history of the superfamily reveals an impressive catalogue of molecular evolution mechanisms that resulted in remarkable functional innovation and specialisation from a relatively small set of components.

## Abstract 2

Comparative genomics and phylogenetic analysis show how a handful of key components have given rise to a profusion of apparatuses involved in important bacterial and archaeal cellular processes (motility, adhesion, protein secretion, and DNA uptake).

## Introduction

New complex forms, functions, and molecular systems arise by the shift in function (co-option) of elements that may have evolved to tackle different adaptive needs [1]. At the molecular level, this involves tinkering with pre-existing molecular structures by diverse processes, including mutation, recombination, and gene fusion and fission [2]. These variants are ultimately subject to natural selection and may eventually become fixed in populations [3]. In Bacteria and Archaea, this is facilitated by the constant income of novel genetic information by horizontal gene transfer [4–6]. Complex adaptations can evolve through a series of small adaptive steps. E.g., metabolic networks evolve stepwise to accommodate novel reactions at their edges [7]. Innovation may also arise by processes of neofunctionalisation or subfunctionalisation following the duplication of genes encoding proteins with multiple functions or the acquisition by horizontal transfer of homologous genetic systems. How these processes shape the evolution of macromolecular complexes remains poorly known.

**Fig 1:**
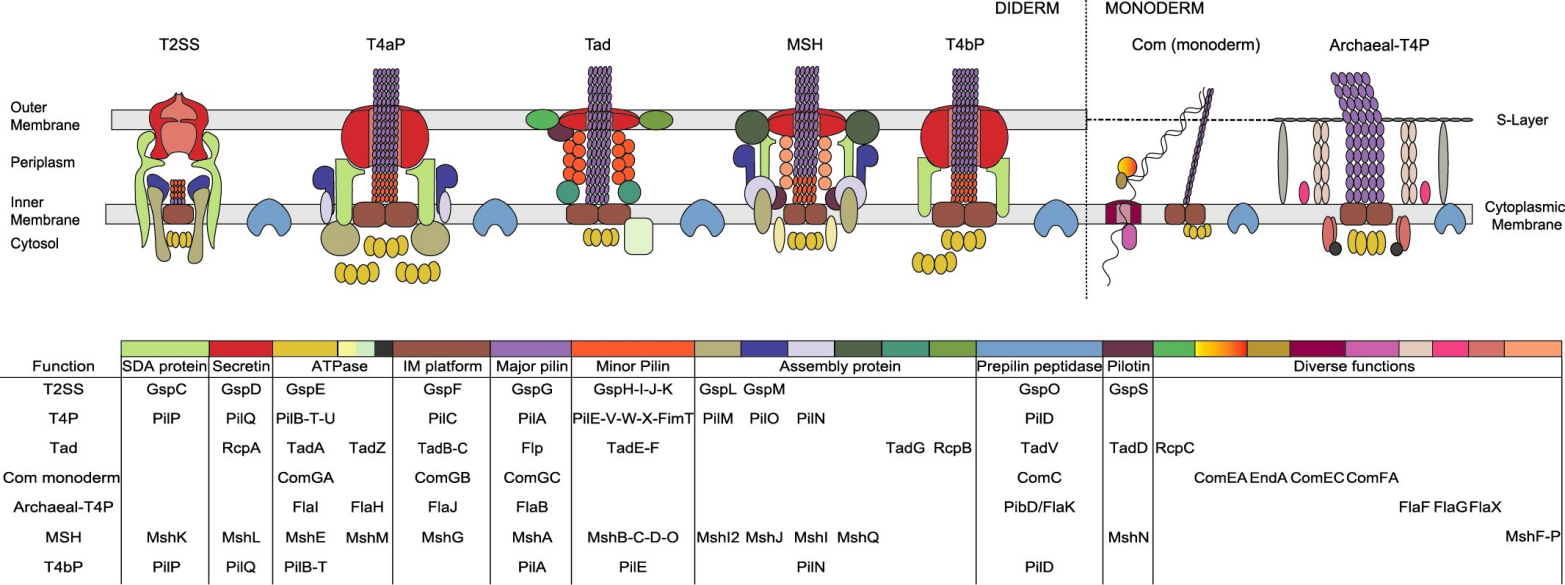
Schematic representation of the different systems and associated genes. Homologous components are represented in the same colour. The table below the drawing indicates the colour code and the name of the different components in each type of system. For the Archaeal-T4P, the representation of the systems is based on the representation of the archaellum, and the genes mentioned in the legend are the names of the genes used in the literature (not the arCOG database’s names). Some systems have multiple homologues of the ATPase, and these are shown as multiple clusters in the figure (with same shape and colour). Archael-T4P, type IV-related pili in Archaea; arCOG, archaeal Cluster of Orthologous Genes; Com, competence pilus; IM, integral membrane; MSH, mannose-sensitive hemagglutinin pilus; SDA, secretin-dynamic–associated; Tad, tight adherence; TFF, type IV filament; T2SS, type II protein secretion system; T4aP, type IVa pilus; T4bP, type IVb pilus.

The appendages of Bacteria and Archaea are striking examples of functional diversification. They are complex macromolecular machineries encoded by many genes and spanning several cellular compartments that can evolve towards novel functions. E.g., the type III protein secretion system (T3SS) evolved from the secretion apparatus of the bacterial flagellum [8], the type IV secretion system (T4SS) from the conjugation apparatus [9], and the type VI secretion system (T6SS) possibly from co-option of phage structures [10,11]. A particularly remarkable illustration of these processes is provided by the type IV filament (TFF) superfamily of bacterial and archaeal systems that include the type II secretion system (T2SS), the type IVa pilus (T4aP), the type IVb pilus (T4bP), the mannose-sensitive hemagglutinin pilus (MSH), the tight adherence (Tad) pilus, the competence pilus (Com), and the type IV-related pili in Archaea (Archaeal-T4P), which includes the archaeal flagella (archaellum). These systems have core homologous components, sometimes in multiple copies, and present similarities in terms of macromolecular architecture throughout Bacteria and Archaea (Fig 1) [12–14]. They include AAA+ ATPases, among which the T4aP PilT is the most powerful molecular motor known [15]; an integral (cytoplasmic) membrane (IM) platform; and a prepilin peptidase that matures a set of specific pilins or pseudopilins (in T2SS) [16]. Bacteria with two cell membranes (diderms) also encode a secretin that forms an outer-membrane pore [17]. Other proteins of these systems are either specific for each system or evolve too fast to allow the inference of homology among all variants.

The TFF nanomachines assemble filaments composed of subunits with an N-terminal sequence motif named class III signal peptide, generically named type IV pilins [14]. These systems are involved in functions typically associated with extracellular pili in Bacteria and Archaea, including adherence, cell–cell attachment, and the formation of biofilms [18–20]. They are exploited by phages for cell infection [21]. T4aP, T2SS, T4bP, and Tad are also important virulence factors in pathogenic Bacteria [22–27]. Nevertheless, and in spite of their homology, TFFs have evolved specific biological functions. T4aP and T4bP allow Bacteria to move by twitching motility (a form of surface movement promoted by repeated cycles of extension–retraction of the pilus) [28,29]. T2SS secrete proteins from the periplasm across the outer membrane [16]. Some Com, T4aP, and Archaeal-T4P facilitate the uptake of extracellular DNA into the cell [30,31]. In Bacteria, these systems are by far the most frequent appendages involved in natural transformation [31], the exception being *Helicobacter*, which use a system derived from a T4SS [32]. Archaeal-T4P include the archaellum involved in motility by rotation of the appendage, extracellular structures involved in sugar uptake (Bindosome or Bas), the UV-inducible pilus of *Sulfolobus* (Ups) involved in establishing cell–cell contacts to enable DNA repair under stress conditions, and several pili with poorly characterised functions [33–35].

The functional diversification of the superfamily is not clade-specific because different types of systems are present in the same clades. This suggests frequent horizontal transfer and/or an ancient origin of the superfamily. T4aP and the Tad pilus can be found in most bacterial phyla [36,37], and Archaeal-T4P in most Archaea [35]. The T2SS, T4bP, and MSH have only been described in diderms [38,39]. The distribution of the TFFs involved in competence is poorly known because different types may be involved in the process, in which they have a necessary but not sufficient role [31], and still keep additional functions associated with motility or adhesion. In summary, the TFF superfamily has diversified into several different functions by co-option processes using a common set of homologous components identifiable across Bacteria and Archaea.

Previous studies dedicated to the evolution of the AAA+ ATPases, Tad, T4aP, and T2SS date from the previous decade [12,37,40,41], when data were scarce and phylogenetic methods less sophisticated. Archaeal systems were studied in detail recently [35,42] but independently of the evolution of bacterial systems. More recent works only briefly studied the phylogenies of some of the components of these systems [43]. Importantly, there is a lack of studies integrating all the systems and all available genomic data, a prerequisite to understand the processes of functional diversification of the superfamily. Here, we identified the typical TFFs and their variants using specific annotation tools on all complete genomes of Bacteria and Archaea. These systems were analysed using phylogenetic techniques to characterise the history of the TFF superfamily, clarify the relationships among its members, and decipher the molecular evolution mechanisms underlying its functional diversification. Finally, we characterised their genetic organisations and how they relate to the rates of horizontal gene transfer. This integrative analysis provided a consistent scenario for the diversification of the superfamily involving processes of gene duplication, fission, transfer, accretion, and mutation.

## Results

### Relations of homology between the key components of the machineries

We started our study by building MacSyFinder models [44] for the identification of TFFs in the complete genomes of Bacteria and Archaea. Briefly, these models give a detailed account of the genetic composition and organisation of the systems. We adapted previously published models of T4aP (including the Com systems of diderms), T2SS, and Tad [39,45], in which we incorporated additional components and stricter rules in terms of genetic composition and organisation to identify TFFs with high stringency for the initial phylogenetic and genomics analyses (S1 Fig). We used the literature to produce equivalent models and associated hidden Markov model (HMM) protein profiles for the Com of monoderms (ComM) and for the Archaeal-T4P. For the latter, we used 66 archaeal Cluster of Orthologous Genes (arCOGs) identified from [35] after a step of reanalysis of the initial 191 arCOGs to remove redundancy. We could not build models for T4bP and MSH systems at this point because too few systems were described in the literature. This work resulted in five initial models, including 154 HMM protein profiles, of which 17 are novel (S1 Table).

**Fig 2:**
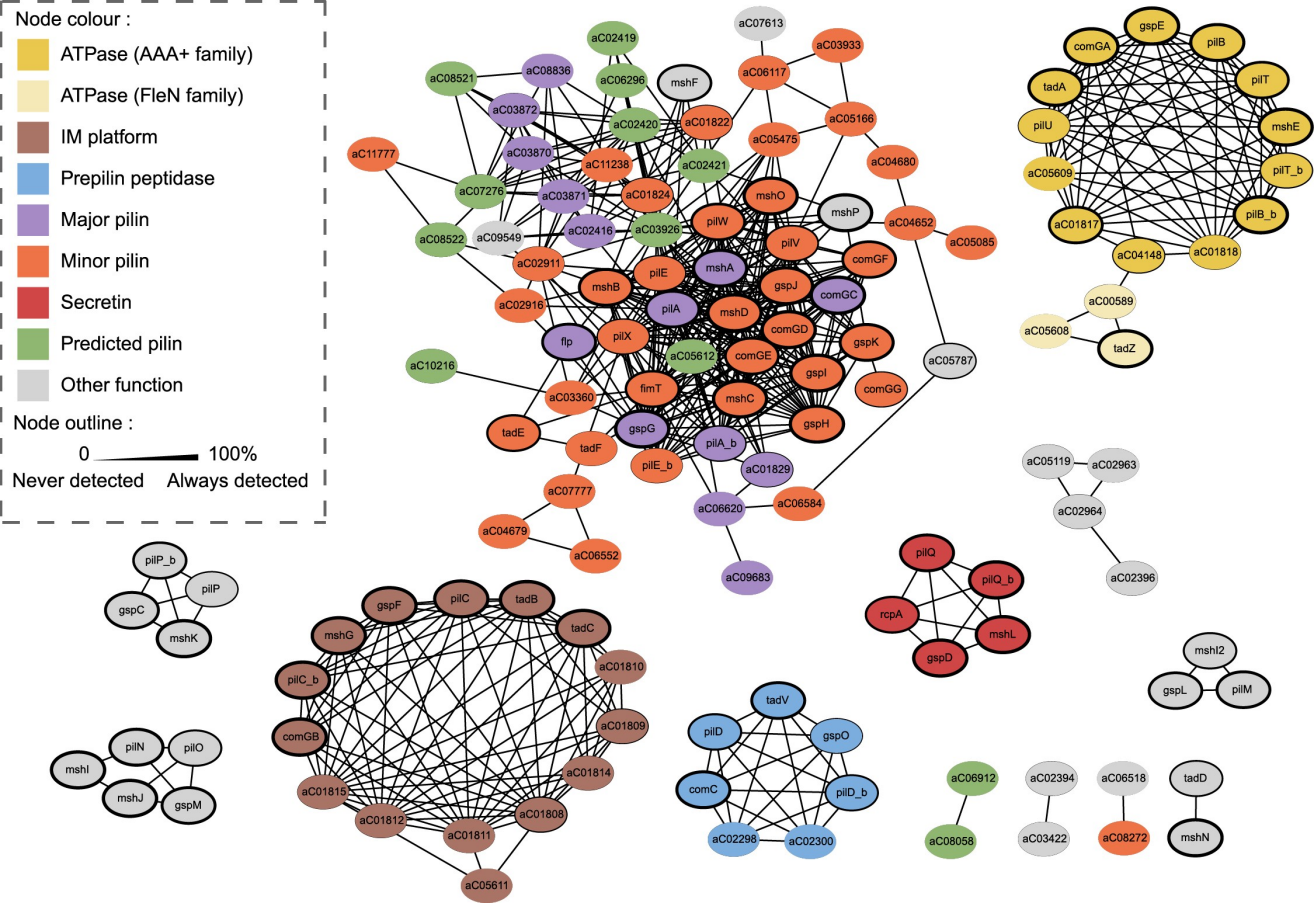
Results of the HMM–HMM alignments (HHSearch) between all the components of the TFF superfamily. The colour of the nodes represents the known or predicted function of the protein. The size of the outlines is proportional to the frequency of the profiles in the detected systems (thicker outlines indicate higher frequencies). Com, competence pilus; HMM, hidden Markov model; IM, integral membrane; MSH, mannose-sensitive hemagglutinin pilus; Tad, tight adherence; TFF, type IV filament.

To establish the relations of homology between the components of the different systems in a precise and homogeneous manner, we made pairwise profile–profile alignments of their HMM protein profiles using HHsearch v3.0.3 [46] (*p*-value threshold of 0.001). These alignments are very sensitive and highlight more distant relations of homology than typical sequence alignment methods [46]. We obtained a graph with 10 components (sets of connected nodes), representing the significant relations of reciprocal similarity between the profiles (Fig 2). The five largest components include the proteins known to be homologous and represent each individual key function: secretins, prepilin peptidases, ATPases, IM platforms, and pilins (major and minor). The ATPase component includes TadZ, a protein from another subfamily of P-loop ATPases (FleN) with an atypical Walker‐A motif that retains ATP binding capacity while displaying low ATPase activity [47,48]. It localises at the pole at early stages of pili biogenesis and functions as a hub for recruiting other Tad pili components, contrary to the ATPases involved in pilus assembly or retraction.

These results establish a precise and extensive network of sequence similarity between the key components of TFFs, systematising previous descriptions. The largest component of the graph includes the major and minor pilins, which are small and very diverse across the TFF superfamily. Their profile–profile alignments suggest they are all evolutionarily related. The remaining components were smaller and usually revealed at most one component per TFF family.

### The phylogenies of the components of the TFF superfamily

The presence of homologues of the major functional components of the TFFs across most types of systems raises the question of how their functional diversification took place from a common ancestor. To study this, we added to the models described above a very simple generic model to identify all systems with three key components (the ATPase, the IM platform, and a major pilin). Accessorily, it also searches for a secretin, absent in monoderms, and a prepilin peptidase, sometimes shared between systems [49–51] (S1 Fig). The search for systems using the MacSyFinder models resulted in the identification of 6,652 systems in 3,700 genomes (1,486 species) (S2 Fig), of which 1,584 were classed as generic systems, reflecting the conservative character of the initial models. This data set was too large to analyse using sophisticated phylogenetic methods and included many systems that were very similar, e.g., from different strains of the same species. We reduced this redundancy by clustering very similar systems. We then picked one representative per cluster, thus preserving most of the diversity of the data set. In this process, we prioritised the inclusion of experimentally validated systems, including MSH (1) and T4bP (5), for which models were not available (see Methods). This nonredundant set contains 309 representative systems (33 T4aP, 47 Archaeal-T4P, 29 T2SS, 5 T4bP, 1 MSH, 31 ComM, 72 Tad, 101 generic) (S2 Table). Hence, the systems used in the subsequent analyses are associated with a (sometimes large) number of other very similar systems that are from the same cluster.

We inferred the phylogeny of each of the five core protein components (AAA+ ATPase, IM platform, major pilin, secretin, and prepilin peptidase) by maximum likelihood with IQ-Tree [52]. We made 10 reconstructions per component with the most thorough mode of topological search to account for the stochasticity of the method. The detailed analysis of key events revealed by these trees can be found in S3 Table (the trees themselves are in S4 Table). The ATPase trees are very well supported at most of the key nodes, they are consistent across replicated inferences, and they clearly separate the different types of systems (S3 Fig). The trees include two system-specific duplication events of the ATPases, one ancestral to the large clade—including T4aP, T4bP, MSH, T2SS, and ComM (PilT/PilB)—and another within a clade of T4aP (PilT/PilU). The IM platform tree also discriminates between types of systems and includes pairs of homologues in Tad (TadB/TadC) and some archaeal systems (S6 Fig). The prepilin peptidase tree is poorly supported and shows scattered distribution of the different types of systems (S8 Fig). Because prepilin peptidases can be exchanged between systems [49–51], we have excluded them from further analyses. The secretin and major pilin trees have some poorly supported branches, but they separate the different systems well. Overall, the protein components’ trees show that the ATPase, the IM platform, and the major pilin are good phylogenetic markers for the evolution of the TFF superfamily. The secretin tree, even if relatively well supported, is less informative for inferring the global evolutionary scenario because the component is absent from monoderms.

### The root of the TFF superfamily

The ATPase tree is the only one that can be rooted because this is the only ubiquitous component with well-conserved homologues in distinct machineries that can serve as external groups [40,41]. We used FtsK as an outgroup to root the tree because it is very conserved, single-copy, present and essential in most bacterial phyla [53,54], and shows little evidence of horizontal transfer [41]. Its closest homologue, HerA, is an archaeal protein from which it diverged concomitantly with the archaeal–bacterial division after the last universal common ancestor [41]. We retrieved the sequences of FtsK from a previous study [9], aligned them with the ATPase sequences of the investigated systems, and inferred a maximum likelihood tree. This tree shows that the FtsK sequences are monophyletic (100% Ultrafast Bootstrap Approximation [UFBoot] support) and branch between two large clades: Tad and Archaeal-T4P on one side (100% UFBoot) and a clade grouping the T2SS, T4aP, ComM, and T4bP on the other side (100% UFBoot) (S4 Fig). The overall rooted topology is very similar to that of the unrooted tree in 8 out of 10 trees (S3 Table). The inclusion of the ubiquitous ATPase of the T4SS (VirB4) as an outgroup with FtsK also showed a split between the archaeal and the bacterial branches of the tree (S5 Fig). This confirms that this ATPase family is also an outgroup of the TFF superfamily. We rooted the trees of the IM platform and major pilin using the root of the ATPase trees because all three proteins showed a consistent split between Tad/Archaeal-T4P on one side and the remaining systems on the other (S6 and S7 Figs).

The analysis of gene duplications provides additional information on the possible roots of the superfamily phylogenetic tree. Placing a duplication event on a tree corresponds to setting as anterior the branch in which the duplication occurred, and as posterior, those of the two paralogues [55,56]. The duplications of the ATPases therefore exclude the root from the group T4aP, T4bP, ComM, MSH, and T2SS. The duplication of the IM platform in the Tad system, also present in some Archaeal-T4P, excludes the root from within these groups. Hence, the analyses of duplication events are consistent with the root as defined above by the tree of the ATPases.

### Producing a concatenate tree

**Fig 3:**
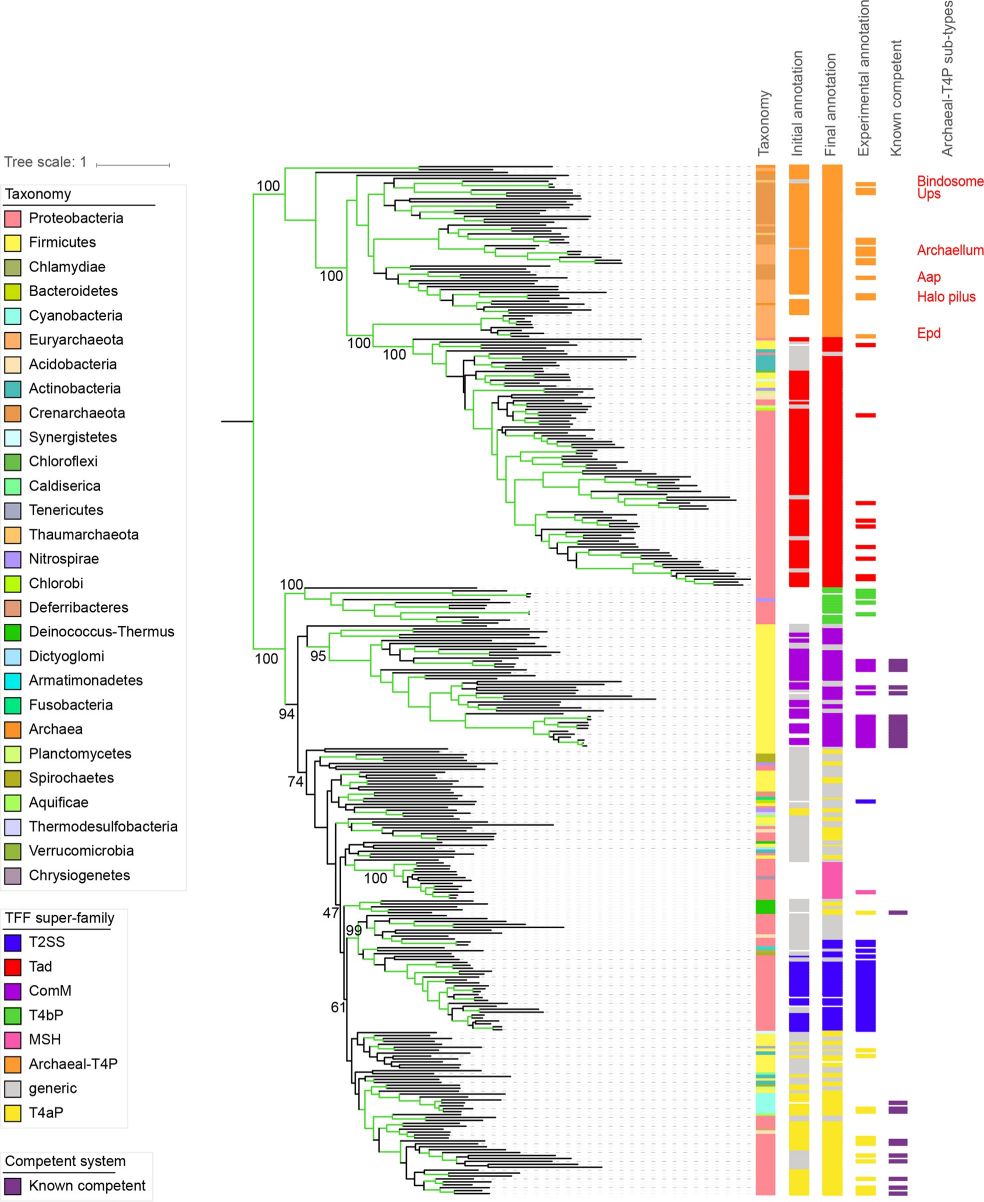
Rooted phylogeny of the TFF superfamily. The tree was built with the concatenate of the IM platform (using TadC) and the AAA+ ATPase (using PilB). The branches are in green if the ultrafast bootstrap is >95%. The supports of the significant nodes are indicated in text. The different coloured strips indicate the classification of the systems with the MacSyFinder annotation (with the initial model and with the final one) and the annotation of the systems in the literature. The systems known to be implicated in natural transformation are indicated in dark purple. Known subtypes of Archaeal-T4P are indicate by text in red. The tree was built using IQ-Tree, 10,000 replicates of UFBoot, with a partition model. Halo pilus indicates two pili characterised in Halobacteria. Aap, adhesive archaeal pilus; Archaeal-T4P, type IV-related pili in Archaea; ComM, competence pilus of monoderms; Epd, EppA dependent; IM, integral membrane; MSH, mannose-sensitive hemagglutinin pilus; Tad, tight adherence; TFF, type IV filament; T2SS, type II protein secretion system; T4aP, type IVa pilus; T4bP, type IVb pilus; UFBoot, Ultrafast Bootstrap Approximation; Ups, UV-inducible pilus of *Sulfolobus*.

Because the ATPase and the IM platform have phylogenetic trees that are broadly consistent (S3 Table) and are the most informative markers of the phylogeny, we computed a phylogenetic tree of their concatenate using a partition model (best model for each gene partition, as computed by IQ-Tree). The major pilin was excluded from the concatenate because it shows less-consistent and less-supported topologies. Concatenation required the use of a procedure to deal with multiple homologues in the same system (to have one marker per component per system). For those present in a few taxa, we chose in each system the protein most similar in sequence to the most closely related systems lacking paralogues (see Methods). For the ATPases, we used PilB because this ATPase is responsible for the assembly of the pilus, which is an essential function in all families, contrary to the function of PilT/PilU (retraction). There was no good argument to pick TadB or TadC platform proteins, and we therefore made 10 phylogenetic reconstructions with each of them in parallel in ATPase/IM platform concatenates. As expected, the best trees (highest log likelihood) for the two TadB/PilB and TadC/PilB concatenates were never rejected by the individual proteins' alignments (*p* > 0.05, Approximately Unbiased [AU test], S7 Table). Furthermore, after correction for multiple comparisons, only two of the 40 comparisons between the individual proteins and the concatenate trees were significantly incongruent (TadB versus the two trees with lowest likelihood obtained for the TadB/PilB concatenate). We present in Fig 3 the highest log-likelihood tree obtained for the TadC/PilB concatenate (the combination of markers with no significant conflict with the gene trees). Overall, these concatenate trees show that the TFF families derived from an ancestral system, which diversified initially into an archaeal system ancestor of Tad/Archaeal-T4P and a bacterial system ancestor of the remaining TFFs.

### The archaeal systems and the emergence of Tad

The ATPase, IM platform, and concatenate trees are broadly consistent with five or six groups within Archaea (Fig 3, S3, S6 and S10), several of which replicate previous findings (groups archaellum, Halo pilus, Epd, ‘Adhesive archaeal pilus’ [Aap], Bas/Ups [35]). All experimentally validated archaella are part of a highly supported clade (100% UFBoot, group 3 in [35]) that is the sister clade to another highly supported clade containing two pili involved in surface adhesion in Halobacteria (Halo pilus, group 2 in [35]). They are sister groups of a clade gathering the Bas, the Ups, and noncharacterised pili from Crenarchaeota and Thaumarchaeaota (group 4 in [35]). Aap cluster with the Halo pilus in the concatenate TadC/PilB and group apart closer to the Bas and the Ups pilus in other trees. The rooted tree shows two basal clades of Archaeal-T4P systems of unknown function, mostly found in methanogens (group 1 from [35], which is separated by the root in our tree).

Unexpectedly, the position of the root places Tad as a system derived from Archaeal-T4P systems. This feature is found in the trees of ATPase, IM platform, and major pilin with high confidence. Furthermore, all these trees showed a monophyletic clade, including the Tad and the ‘EppA-dependent’ (Epd) pilus (clade ‘Epd-like’), whose major pilins have similarly short sequence lengths when compared to the others from Archaeal-T4P (S7 Fig). Both Epd-like pili and Tad have two homologous genes encoding the IM platform, suggesting that their common ancestor already contained them both. We examined the domain structure of these two genes and found that each has one ‘T2SSF’ domain (PFAM domain PF00482), whereas most other Archaeal-T4Ps have two such domains and longer IM platform proteins. This strongly suggests that TadB and TadC were derived from an ancestral event of gene fission and not a duplication as previously suggested. To confirm this observation, we aligned the TadB and TadC profiles with the archaeal IM platforms containing two T2SSF domains. In these cases, TadC aligned best with the N-terminal domain, while TadB aligned best with the C-terminal domain of the archaeal proteins. To further test the gene fission scenario, we made a tree using the concatenate of TadC and TadB, and this tree was similar to the tree of the concatenate (S4 Table). Finally, the Tad systems have a protein, TadZ, that has significant HMM–HMM profile alignments with Archaeal-T4P components (arCOG00589 and arCOG05608), including those from the Epd-like clade (group 1 from [35]), but not with profiles from the bacterial systems. Altogether, these results strongly suggest that an ancestral Archaeal-T4P harbouring two genes encoding the IM platform diversified into Epd-like systems in Archaea and was transferred horizontally, apparently only once, to Bacteria, leading to the extant Tad systems.

The transfer of the system from Archaea to Bacteria was very ancient. Tad systems were frequently transferred among Bacteria since then (see below), and it is not possible to infer the precise bacterial taxa that acquired the original system. However, the Tad systems at the basis of the clade are from Proteobacteria in 18 out of 20 concatenate trees, often with very good support (S3 Table). The two odd concatenate trees place Firmicutes at the base of the Tad clade but with very low support. This suggests that the ancestor of the Tad system was acquired by a diderm bacterium, and the accretion of the outer-membrane, pore-forming secretin to the Tad system may have been the founding event of these systems.

### The diversification of the bacterial TFF superfamily

The other major clade of the TFF superfamily only has bacterial systems (T4aP, T4bP, ComM, MSH, T2SS). The vast majority of the concatenate and component trees place T4bP at the basal position in the clade (in the others, some generic systems take this position). This is followed by a split between ComM on one side and T4aP, MSH, and T2SS on the other. T4aP are polyphyletic in all the phylogenetic reconstructions, showing a few clusters with the experimentally validated systems (Fig 3). Some of these systems are in monoderms such as Firmicutes and Actinobacteria, as previously observed [14,57]. MSH and T2SS are both clearly distinct and derived from the T4aP. The MSH system falls in a highly supported clade (100% UFBoot) with other systems of very similar gene composition. Intriguingly, all MSH loci lack a prepilin peptidase. They may use a protein from another system because MSH were systematically present in genomes with T2SS, T4aP, or Tad, which encode a prepilin peptidase. Systems previously identified as T2SS show two exceptions to monophyly. First, the position of chlamydial T2SS next to the other T2SS is highly supported in the ATPase and in the concatenate tree (>95% UFBoot) but not in the trees of the secretin, major pilin, and IM platform. This suggests a chimeric origin for this system in which different components were recruited from different types of systems. Second, the so-called T2SS of Bacteroidetes (represented by *Cytophaga*, [58]) always cluster with T4aP and away from the remaining T2SS.

The key early event in the ATPase trees of the Bacteria-only TFF large clade was the amplification leading to the paralogues PilB (the assembly ATPase) and PilT (the retraction ATPase). This event appears as a simple duplication at the base of the tree in certain of the ATPase trees but also shows more complex scenarios in others (S3 Table). In the PilB part of the ATPase tree, T4bP is basal, and the other systems are regrouped with T4aP. This scenario is consistent with that of the secretin tree, in which if one places the root between T4aP and T4bP, one finds T2SS deriving from a T4aP system, as in the PilB trees. This is also sustained, albeit with low support, by the major pilin tree, in which one finds at basal positions T4aP and T4bP. The presence of PilT in the early stages of evolution of the TFF superfamily could be an indication that the most ancient systems already had ATPases specialised in pilus retraction.

One of the most interesting functions of the superfamily, from the evolutionary point of view, is the involvement of some of its systems in natural transformation. The ComM system is commonly found in Firmicutes, even if it is unclear whether it is always involved in transformation. It is monophyletic in all the phylogenetic reconstructions we made, usually with very high support (≥95%). In the concatenate trees, ComM branches apart from a group gathering T4aP, MSH, and T2SS after the divergence with T4bP. The trees of individual components show similar scenarios once one accounts for the effects of the ATPase paralogues and for the low support of some parts of the IM platform trees. In summary, these results suggest that ComM arose early and only once in the history of the TFF superfamily. The T4aP systems experimentally linked to natural transformation in diderms were systematically identified as T4aP and also tend to cluster together in the tree.

### TFFs are ubiquitous in the prokaryotic world

We used the best concatenate tree, rooted using the information of the rooted ATPase tree, to class the numerous generic systems that we had previously identified. We assumed that clades in which all systems were either generic or of a single type (of which at least one was validated experimentally) could be tentatively assigned to that type. Generic systems in clades lacking experimentally validated systems were left unassigned. Only two types of systems were paraphyletic in the tree—T4aP and Archaeal-T4P—and were thus treated differently. T4aP was split in a few monophyletic clades, and systems within each clade were reassigned using the method above. The Archaeal-T4P systems, from which Tad derives, can be easily distinguished from the latter and thus reassigned using a taxonomic criterion. This analysis significantly clarified the systems’ assignment (compare S2 Fig with S11 Fig): 1,795 out of the 2,031 generic systems were reassigned to classical systems, mostly T2SS (479) and T4aP (748).

We used these tentatively assigned systems to produce more sensitive MacSyFinder models. First, we changed the HMM profiles to account for the genetic diversity introduced by the reassigned systems. Second, we created models to detect T4bP and MSH because we now had a much larger number of examples of these systems. Finally, we searched for genes systematically associated with the systems’ loci in a neighbourhood of ±20 genes that were not matched by any of the HMM profiles of the models. We clustered the proteins by sequence similarity and analysed the largest families. This ‘guilt-by-association’ approach failed to show other proteins systematically associated with a particular type of system (S5 Table), suggesting that our models already encompass their most frequent components. This process resulted in more sensitive models that accounted for all known types of systems and correctly identified the 94 experimentally validated systems of Bacteria analysed in S2 Table, except the T2SS of Chlamydia and Bacteroidetes (shown above to be peculiar).

**Fig 4:**
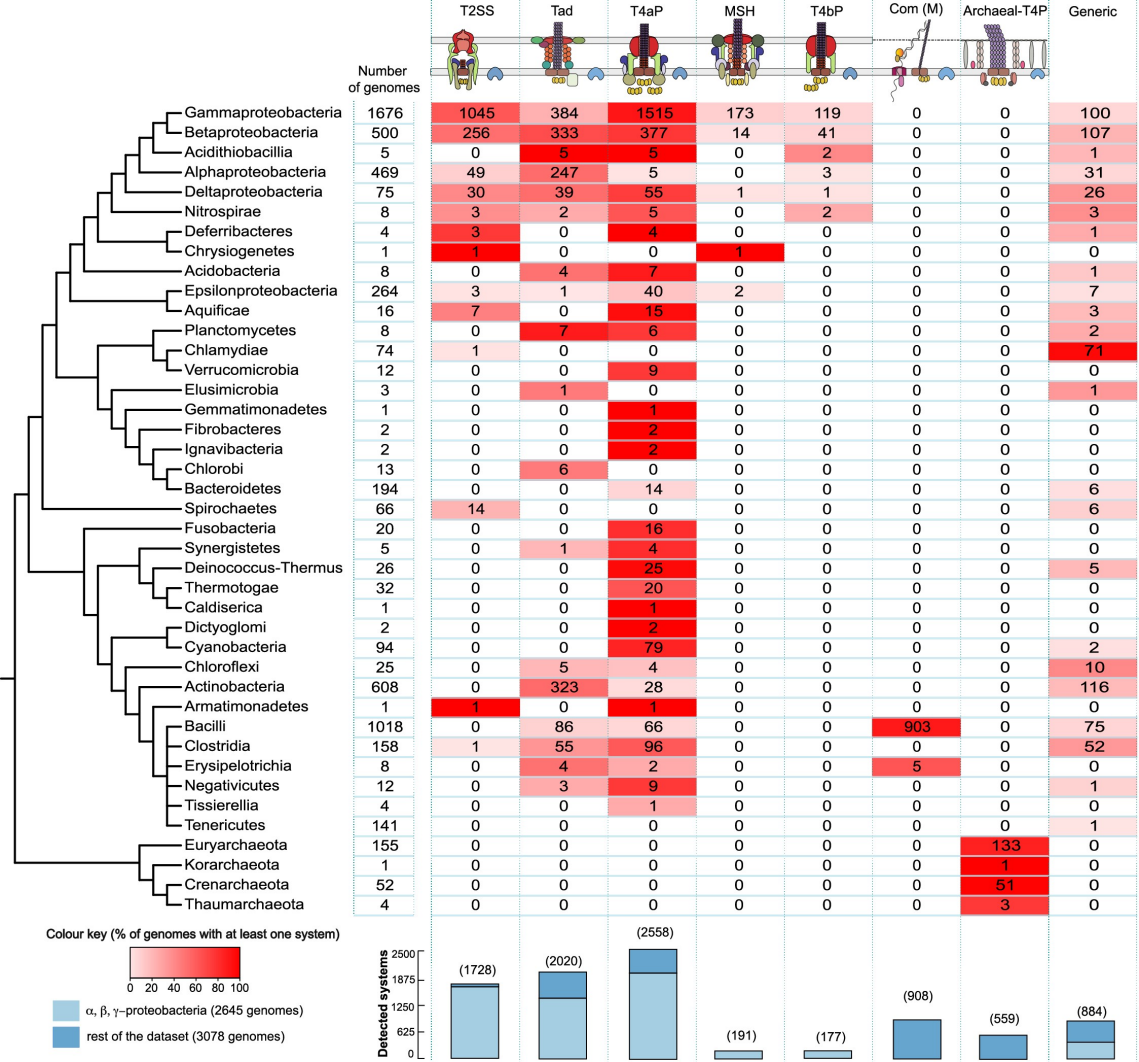
Taxonomic distribution of the systems in Bacteria and Archaea obtained using the final models. Cells indicate the number of genomes with at least one detected system. The cell’s colour gradient represents the proportion of genomes with at least one system in the clade. The bar plot shows the total number of detected systems. The bars are separated in two categories: Alpha-, Beta-, and Gamma-proteobacteria versus the other clades. The cladogram symbolises approximated relationships between the bacterial and archaeal taxa analysed in this study. Archaeal-T4P, type IV-related pili in Archaea; Com, competence pilus; ComM, Com in monoderms; MSH, mannose-sensitive hemagglutinin pilus; Tad, tight adherence; T2SS, type II protein secretion system; T4aP, type IVa pilus; T4bP, type IVb pilus.

Using the improved models, we found 9,026 systems within 4,610 genomes, including 1,728 T2SS, 2,021 Tad, 2,558 T4aP, 908 ComM, 559 Archaeal-T4P, 177 T4bP, 191 MSH, and 884 generic systems (Fig 4, S6 Table). A few systems classed in a given type with the initial conservative models—14 T2SS, 10 T4aP, 5 Tad, 1 ComM—are classed as generic with the new models. However, the inverse is much more frequent because we reclassified 1,114 generic systems as 1,408 T4aP, 338 T2SS, 670 Tad, 4 ComM, and 226 Archaeal-T4P. The large number of generic systems reassigned to T4aP is not surprising because these systems are encoded in multiple loci, are very diverse, and are present in several clades in the tree. This makes them harder to detect using the initial model. The many reassignments of generic systems as T2SS reflect a posteriori the excessive stringency of our initial model based on existing knowledge of systems in Proteobacteria and the existence of T2SS with little or no experimental evidence in other phyla. The reassignment led to identification of T2SS in a much broader set of taxa, including Armatimonadetes, Deferribacteres, Clostridia (from a clade known to contain diderms, further supported by the presence of a secretin), Spirochaetes [59], and Aquificae. We also observe many new Tad systems in Elusimicrobia, Actinobacteria, Bacilli, and Clostridia (Fig 4 versus S2 Fig). Our phylogenetics-driven approach for designing new models allowed us to detect diverse putative MSH and T4bP. These systems were so far only described as such in Gamma-proteobacteria, but we identified them also in Chrysiogenetes and Epsilon-proteobacteria for MSH and in Acidithiobacillia and Nitrospirae for T4bP.

In certain cases, the phylogenetic annotation identified some systems that we missed using the improved, models and provides information to explain the large number of generic systems in certain clades. The T2SS in Chlamydiae [60] are close to the other T2SS for several phylogenetic markers but are classed as generic because they apparently lack homologues of the minor pilins and the assembly proteins GspLM [60]. Many of the systems of Actinobacteria remain classed as generic systems. A large fraction of them could be classed as Tad by proximity to experimentally validated systems in the phylogeny, but they lack identifiable homologues of some usual components such as the minor pilins and TadC (their TadB does not contain two domains like those of homologues in some Archaea, showing this is not the result of a gene fusion).

### Genetic organisation is associated with differences in rates of horizontal transfer

**Fig 5:**
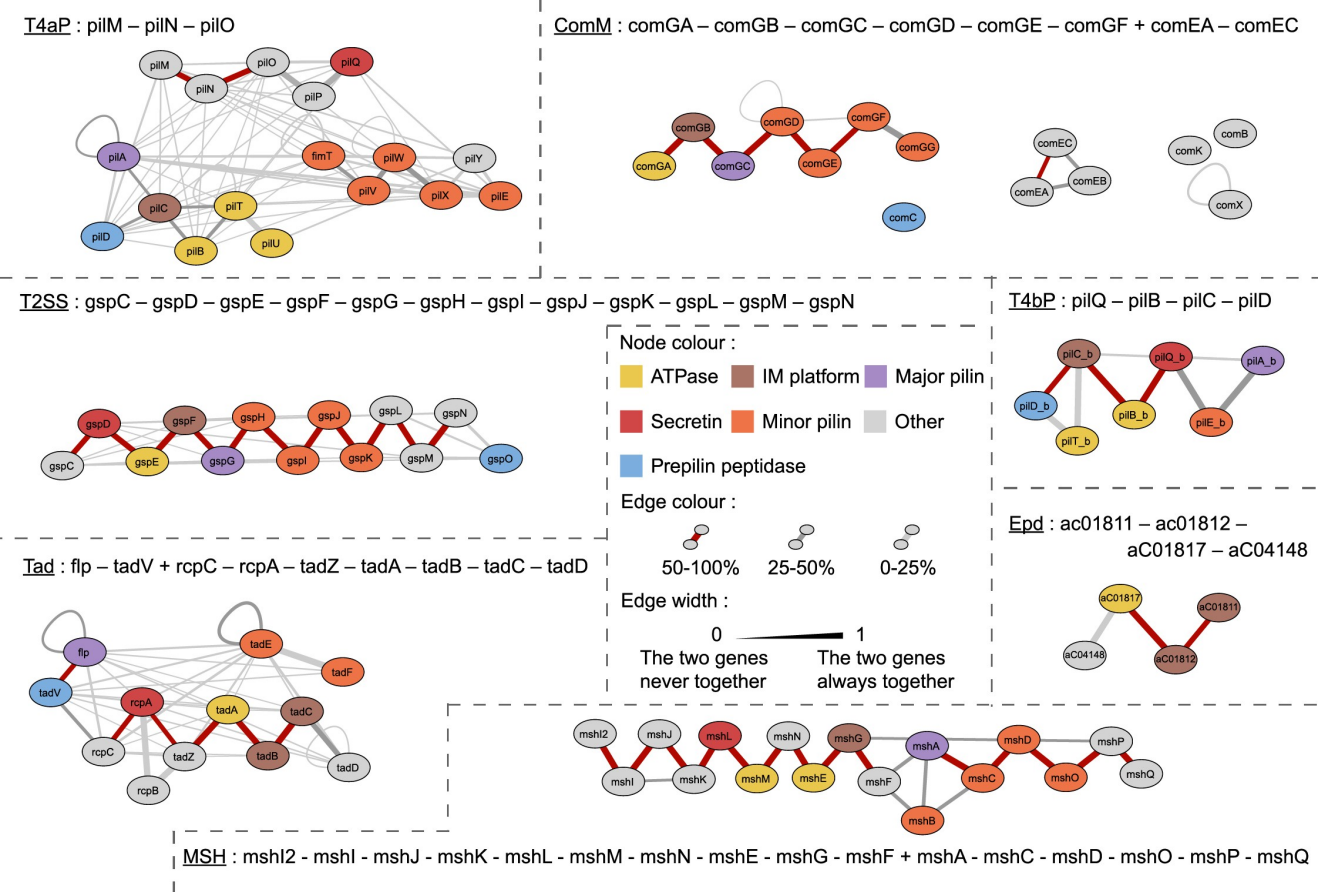
Genetic organisation of the detected systems. For each detected system (those indicated in Fig 4), the edge width represents the number of times the two genes are contiguous divided by the number of times the rarest gene is present in the system. The colour of the edge represents the number of times the two genes are contiguous in the system divided by the number of systems. Com, competence pilus; ComM, Com in monoderms; Epd, EppA dependent; IM, integral membrane; MSH, mannose-sensitive hemagglutinin pilus; Tad, tight adherence; T2SS, type II protein secretion system; T4aP, type IVa pilus; T4bP, type IVb pilus.

The systems differ strikingly in terms of genetic organisation (Fig 5). ComM and T4aP are usually found in multiple loci, whereas MSH and Tad are almost exclusively encoded in a single locus. This characteristic further contributes to set MSH apart from the remaining T4aP. Hence, as systems diverged, their genetic organisation also changed. To detail the prototypical genetic organisations of each type of system, we built a graph on which nodes represent components and edges link components that are encoded contiguously in the genome. The edges are weighted by the frequency of contiguity: genes that are systematically contiguous are linked by thick edges. This graph quantifies the prevailing genetic organisations for most types of systems (Fig 5). The Archaeal-T4P show very diverse genetic organisation, presumably because they include very different systems (S12 Fig). The representative archaella systems show more conserved genetic organisation [35,42] (S13 Fig). Interestingly, the genetic organization of the key components of Epd is very similar to the Tad, presumably pre-dating Tad's ancestor transfer to Bacteria: the two IM platform genes are contiguous and followed by the major ATPase and the secondary one (TadZ in Tad and FlaH [arCOG04148] in Epd) (see Fig 5).

The patterns of genetic organisation of the homologous components differ between systems. In general, pilins are encoded in a single locus but can vary in their colocalisation with the rest of the genes: they can be apart (T4aP), at the edge of the locus (ComM, Tad), or in the middle (T2SS, T4bP). In Archaea, all cases were found. Interestingly, many duplicated genes tend to be contiguous, e.g., *pilTU* (ATPases). This is consistent with models suggesting that duplication processes often produce tandem duplicates [61]. The variability between types of systems and the conservation within types suggest that genetic organisation is under selection within types but changes rapidly upon functional innovation.

**Fig 6:**
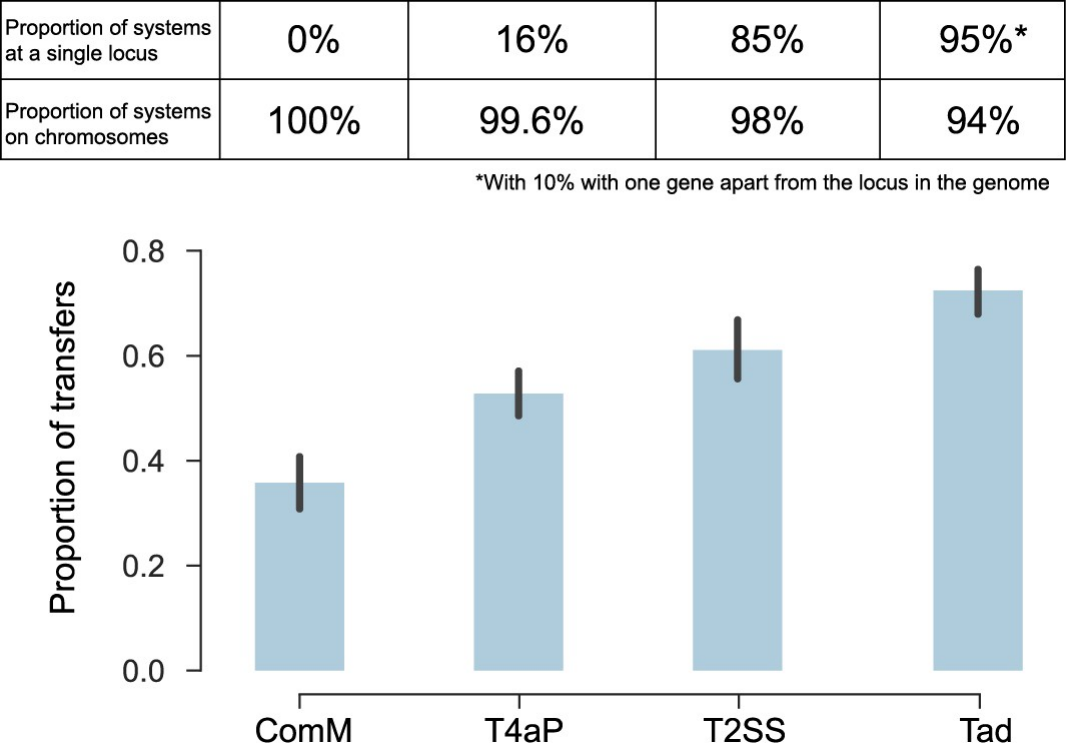
Association between organisation and horizontal transfer of the different systems. For each system, we compared the subtree of the systems with the 16S tree of the same species using ALE v0.4 to obtain the proportion of transfers. The panel above the graphic indicates the proportion of systems in a single locus and the proportion of systems on chromosomes (the others being found on plasmids). ComM, competence pilus of monoderms; Tad, tight adherence; T2SS, type II protein secretion system; T4aP, type IVa pilus.

The genetic organisation of the loci can also reflect the action of horizontal gene transfer. If the systems are often gained or lost within lineages, as was shown for Tad [62] but much less so for the archaellum [42], then systems encoded in a single locus are much more likely to be successfully transferred because all the necessary genetic information can be transferred in one event [63]. Systems scattered across the genomes cannot be transferred in a single event (although parts of the system can presumably be exchanged if the recipient genome encodes a system with similar genetic organisation). We thus hypothesised that single-locus systems are more likely to undergo horizontal gene transfer. To test this hypothesis, we compared the phylogenetic tree of each system, i.e., a subtree of the larger phylogenetic reconstruction, with a maximum likelihood tree of the 16S rRNA sequences of the species carrying the systems (S14 Fig). We excluded the archaeal systems from these analyses because their loci are harder to define precisely (sometimes scattered and multiple systems per genome) and their functions are still poorly delimited in most cases (complicating the definition of the clade to use in the analysis). We found that systems encoded systematically in a single locus are more frequently transferred than those encoded in several loci (Fig 6). These results are reinforced by the analysis of the frequency with which systems are encoded in plasmids, which closely follows the trends observed for the frequency of transfer (highest in Tad and lowest in ComM; Fig 6). The contrast is especially interesting between the Tad and T4aP systems that are both present in many different clades and are encoded almost exclusively in one locus (Tad) or many loci (T4aP). This association between rates of transfer and organisation suggests that systems that are frequently gained and lost endure a selective pressure for being encoded in a single locus.

## Discussion

We used comparative genomics and phylogenetics to produce models and protein profiles that identify TFFs in the genomes of Bacteria and Archaea. The final models classify most systems and assign them classifications that are consistent with the phylogenetic analysis. Some discrepancies persist. They can be due to systems very divergent from the models (see below) or to the presence of inactive and partly deleted loci (remnants of formerly functional systems). The models are publicly available and provide a significant advance relative to our previous work because they are more sensitive and cover more types of systems (Archaeal-T4P, ComM, MSH, and T4bP). We used them to quantify the frequency and taxonomic distribution of the different systems and found that every inspected phylum of Bacteria and Archaea has TFFs from one or several families. Some of these are widespread (e.g., T4aP, Tad), whereas others (MSH, T4bP) are abundant in Proteobacteria but absent from most other phyla. With the exception of archaella, most archaeal systems are poorly characterized. When known, they tend to have diverse functions, components and genetic organization. Further experimental study of these systems is required to produce reliable MacSyFinder models for each of them.

Our approach may be regarded as conservative. First, some components of the systems were excluded from phylogenetic analyses because they were not sufficiently conserved in terms of amino acid sequence. The minor pilins are a particularly important set of proteins that were ignored because they produced short and very poor multiple alignments across systems. Second, the models were built based on experimentally validated systems and using information from monophyletic clades of a given type. If the systems were described in few species or in a small number of phyla, our ability to identify them is limited, especially when they are very different from known systems in terms of gene repertoires and protein sequences.

These limitations may explain why our improved models classed the T2SS of Chlamydiae as generic: they carry few components, and they have different origins. This may result from the impact of the peculiar developmental cycle and intracellular lifestyle of *Chlamydia* on its envelope [60]. In other cases, systems may actually differ from the descriptions in the literature. This is probably the case of the so-called T2SS of Bacteroidetes. This system is involved in protein secretion [58] but consistently branches apart from T2SS in all analyses of the phylogenetic markers. The major pilin of this system is very divergent compared to major pseudopilins from Proteobacteria. Our analysis raises the exciting possibility that it might represent a novel type of protein secretion system derived from the T4aP independently of the T2SS.

All trees show that the widely studied T4aP systems are very diverse and form several different clades in the tree, whereas the one with the PilU ATPase, the most widely studied, accounts for a minority of the identified systems. Most of the other T4aP are poorly characterised and may represent systems with novel properties. Finally, the results obtained with the final improved models showed few systems identified as generic. This suggests that there may be few novel families of systems to be discovered in the superfamily that contain the three key components (ATPase, IM platform, major pilin) and are present in the phyla represented in the genome database. On the other hand, the diversity of certain types of systems—such as the T4aP and the Archaeal-T4P—may still reveal surprising novel functionalities.

**Fig 7:**
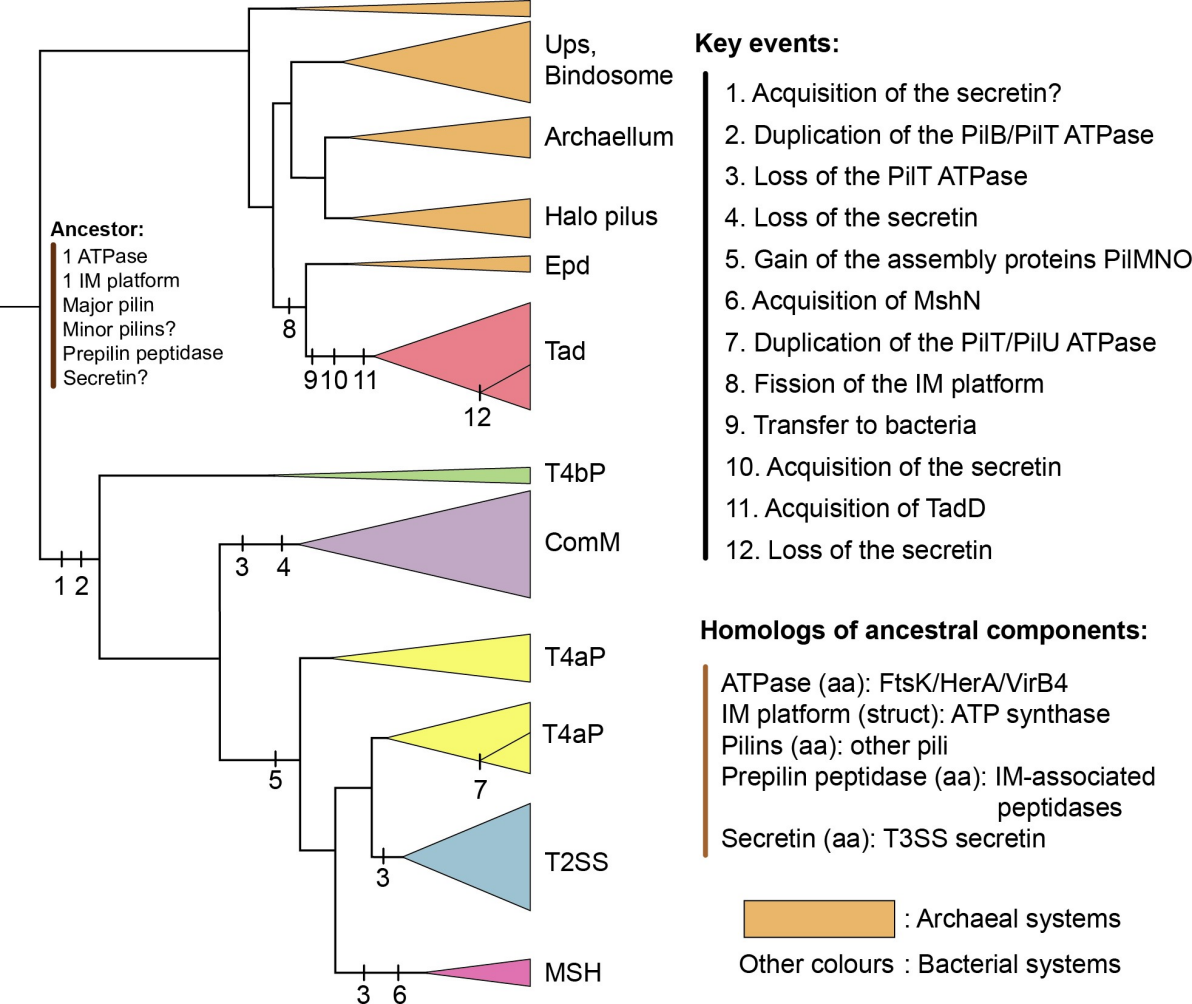
Evolutionary scenario of the TFF superfamily. The tree was based on the information of the trees of the concatenate and simplified to highlight the key clades and events. The colour of the triangles indicates the type of the systems. Each vertical bar on the branch indicates a numbered evolutionary event, whose details are specified under the corresponding number in the list ‘Key events’. The hypotheses for the composition of the last common ancestor of the TFF superfamily are indicated at the root, and the distant homologues of these systems are indicated in the list ‘Homologous of ancestral components’, in which homology was observed by sequence (‘aa’) or structural (‘struct’) similarity. Halo pilus indicates two pili characterised in Halobacteria. Aap, adhesive archaeal pilus; Epd, EppA dependent; IM, integral membrane; MSH, mannose-sensitive hemagglutinin pilus; Tad, tight adherence; TFF, type IV filament; T2SS, type II protein secretion system; T3SS, type III protein secretion system; T4aP, type IVa pilus; T4bP, type IVb pilus; Ups, UV-inducible pilus of *Sulfolobus*.

The phylogeny of the key components of the TFFs revealed an initial split between archaeal and bacterial systems, suggesting that these structures may have pre-dated the last common ancestor of all cellular organisms (Fig 7; see also S8 Table). This ancestral system presumably had one ATPase for its assembly (the function performed by PilB in T4aP), an IM platform, pilins, and a prepilin peptidase. Among these key components, only the ATPase has identifiable sequence homologues outside the superfamily, but the other components have distant sequence or structural homologues that suggest they may pre-date the last common ancestor of all TFFs. The PFAM domain of the prepilin peptidase of TFF belongs to the PFAM clan CL0130 with other signal-peptide inner-membrane–associated peptidases, several of which are found in Bacteria, Eukaryotes (the presenillin family proteases), and Archaea [64]. The protein profiles of the integral membrane platform match those of some ATP-binding cassette (ABC) transporters, and the protein is structurally very similar to one of the V-type ATP synthase subunits [65]. The platform may thus have been co-opted from these ubiquitous membrane-associated systems. The small size and rapid evolution of the pilins preclude the tracing of their evolution at deep time scales. Fast evolution of pilin globular domains may be associated with the variability of essential inner-membrane components that promote pilin targeting to the assembly site or connect the inner- and outer-membrane subcomplexes [66,67]. It is also difficult to determine whether there were other components in the ancestral system of the superfamily because they either evolve swiftly or are present in only a small number of systems. A recent study showed that a minimal set of eight genes was sufficient for the assembly of the T4aP of *Neisseria meningitidis* [68]. Four of them, PilMNOP, are essential for the assembly but are lacking in our list of ancestral genes because their homologs were lacking, or very rare, in genes neighboring T4bP, ComM, Tad, and Archaeal-T4P. They were found in MSH and T2SS (Fig 2, S5 Table), suggesting that they arose more recently and that other systems do not require these proteins for assembly (Fig 7; S8 Table). In short, our results are consistent with the idea that the ancestral system was able to energise its assembly and build up a pilus with matured pilins on top of an assembly platform, the basic molecular architecture of extant systems.

Our results and previous data on the genetic composition and organisation of archaeal systems [35] reveal processes of functional diversification leading to families of different functions. The *Sulfolobus* genus alone counts systems from four of the seven different Archaeal-T4P types studied experimentally (Aap, Bas, Ups, and archaellum). Even though horizontal transfers might be frequent among Archaea, our approach places the root of Archaeal-T4P within systems of methanogens from the Euryarchaeota phylum (group 1 of Makarova and colleagues [35]), and this is consistent with a proposed rooting for the archaeal tree of life within methanogens [69]. Further experimental work is needed to elucidate the functions of these Archaeal-T4P.

The archaeal origin of Tad is consistently suggested by the rooted phylogenetic analyses and the specific shared characteristics of pilins, the IM platform, and TadZ-like proteins in Tad and Archaeal-T4P (S8 Table). The literature often classes Tad pilus as T4bP [70]. Our study shows that these systems are very different in terms of components, genetic organisation, and evolutionary origins. This is in accordance with recent works proposing to clearly separate Tad from T4bP and to name them as T4cP [43]. The Epd-like systems share the closest ancestry with Tad systems among the entire TFF superfamily and are the ones with more similar genetic organization of the key components. They were only characterised in *Methanococcus maripaludis*, in which they are involved in surface attachment, a trait they share with the Tad pilus [71]. A striking trait of Tad (and Epd) is the systematic presence of two genes (*tadB* and *tadC*) encoding the IM platform. This has been regarded as the result of a gene duplication [37], but the size, domain content, and sequence similarity of these genes are more parsimoniously explained by a gene fission event, e.g., by a mutation integrating a stop codon within the ancestral gene. This produces a complex evolutionary scenario: the original IM platform was probably the result of an internal gene duplication event that pre-dated the last common ancestor of the TFF superfamily and is present in most systems. In the Epd and Tad clades, this was followed by a fission event that resulted in two tandem homologous genes. In some Tad systems of Actinobacteria, one of these components (TadC) was lost. The adaptive relevance of these successive events in the light of emerging structural data could be an interesting topic of future research.

The secretin tree provides some information about the process of transfer of the ancestral Tad to Bacteria. It places Tad’s secretin within those of T4aP systems with high confidence and typically close to Proteobacteria. This suggests that the co-option of the secretin upon transfer of the ancestor to a diderm was the founding event of Tad systems. It occurred at a time when most types of systems (T4aP, T4bP, ComM, and possibly MSH and T2SS) were already in place. Tad’s secretin makes a monophyletic clade in the tree, suggesting that the accretion of the secretin to this system only happened once. Interestingly, it has been shown that TadD is essential to the assembly of the Tad secretin in *Aggregatibacter actinomycetemcomitans* [72]. While it was originally thought that TadD had no homologues in the other TFFs, we observed that it has a homologue in MSH systems (MshN, Fig 2). Further work will be needed to determine if the acquisitions of the secretin and TadD are linked or result from independent co-option events. If the scenario of a single, ancestral secretin acquisition in Tad is correct, then the adaptation of Tad to monoderms, which occurs at several places independently in the tree, involved the loss of the secretin. This event of loss seems very common because it is also found once in the initial evolution of ComM and several times at the emergence of T4aP of monoderms. Finally, the large taxonomic distribution of Tad, in spite of its relatively recent origin, is in agreement with the high frequency of horizontal transfer observed for this system.

Secretins were co-opted on multiple occasions in the TFF superfamily. Co-options of a secretin from other systems are very common. They were observed multiple times in the evolution of the T3SS (e.g., from Tad and from T2SS) and in filamentous phages [8]. In this respect, it is interesting to analyse the six TFFs with secretins (3 T4aP, 2 Tad, and 1 T2SS) in Firmicutes with an outer membrane (Halanaerobiales and Negativicutes). Four of these systems group with the TFFs of Firmicutes, and two were more closely related to systems from diderms. Their secretins were placed in the tree with proteins from the same TFF family of diderms and lacked distinctive domain architecture. There is thus no evidence that secretins were co-opted to adapt to the membrane of diderm Firmicutes, possibly because the ancestors of Firmicutes were diderm [73].

The T4bP is the most basal system among Bacteria. Subsequently, a split separated ComM from T4aP, and the latter then diversified into T2SS and MSH. Recent works suggest that the last common ancestor of Bacteria was a diderm [73]. Our analysis shows that Tad, T2SS, and T4aP are monophyletic clades in the phylogeny of the secretin, in agreement with previous works [74], suggesting that there was little transfer of the secretin between systems. If one roots the secretin tree between T4bP and T4aP, as in the concatenate trees, then it largely recapitulates the tree of the TFFs concatenate (except for the position of Tad). This is in line with a very ancient acquisition of the secretin by the TFF superfamily. Because T4bP are the most basal systems in the tree and are only found in diderms, this strongly suggests that the original bacterial system had a secretin and was present in a diderm.

Until recently, it was thought that only systems encoding PilT were capable of pilus retraction. This would suggest that the ability to retract the pilus resulted from the neofunctionalisation of one of the copies (PilB in T4aP) of the protein at the moment of the duplication of the ATPase, leading to PilB/PilT in T4P. Surprisingly, it was recently shown that the Tad system of *Caulobacter crescentus* is also able to retract the pilus [43], and there is evidence that the PilB ATPase is implicated in the process [75]. As these authors, we could not identify any orthologue of PilT in this system, in agreement with a PilT-independent retraction of the pilus. It is possible that the original ATPase of the ancestor system of T4bP and T4aP could perform both activities—assembly and retraction/disassembly—and that the duplication resulted in subfunctionalisation of these functions when both PilB and PilT were present. Interestingly, the T4aP in *Vibrio cholerae* was shown to retract with low speed in the absence of PilT [76], even if *pilT* mutants have extremely low [76] or undetectable rates [77] of DNA uptake. This would contribute to explain how ComM, devoid of PilT, could retract a filament carrying DNA in *Streptococcus* [78] and how nontypeable *Haemophilus* are able to uptake DNA using a T4aP that lacks PilT [79], whereas PilT mutants are defective in transformation in several Bacteria [77]. It was also previously suggested that pilus retraction of the Archaeal-T4P might be required to explain some archaeal communities’ behaviour, like transition from sessile to swimming stages [80]. Some overlap between the functions of PilT and PilB might also explain why PilT is frequently lost (e.g., in T2SS and ComM). In line with the specialisation of the roles of this family of ATPases following duplication, a recent study showed that PilU improves retraction in high friction environments, whereas PilT is sufficient for motility in free solution [81]. It is tempting to speculate that other, rarer duplications of these ATPases are involved in further specialisations of retraction functions.

The increase in the diversity of systems able to retract the pilus opens the possibility that many other pili could be involved in natural transformation because the role of the pilus is to attach the DNA and bring it to the cell surface. Actually, a number of arguments are in favour of the hypothesis that the ancestor of the bacterial systems, or even the last common ancestor of the superfamily, might have been able to facilitate natural transformation. First, ComM, Tad, and T4aP have been associated with this mechanism. Second, the predicted repertoire of genes of the last common ancestor of these types of systems could suffice for DNA attachment and retraction towards the cell envelope necessary for transformation. Third, systems that emerged within Archaea are able to facilitate transformation. The Ups pilus in *Sulfolobus* is highly expressed under UV light, mediates cell aggregation, and facilitates natural transformation mediated by the independent Crenarchaeal system for exchange of DNA (Ced) [82,83]. A Tad locus (archaea-derived, like all Tad) from *Micrococcus luteus* has recently been shown to be required for natural transformation [84]. We identified this system within the Tad clade, and its gene repertoire includes a single ATPase. Fourth, it has been previously shown that other key components of the transformation machinery—DprA and ComEC—are widespread across Bacteria [31,85]. The DNA uptake machinery required for transformation is encoded in many Bacteria that were never shown to be naturally transformable [86,87]. E.g., *Escherichia coli* and other enterobacteria contain functional T4aP genes coregulated with the competence machinery [66,88], which are required for natural transformation [89]. If many TFFs have the same ability, then a vast majority of Bacteria could potentially be naturally transformable. Interestingly, ComM and T4aP systems known to be involved in natural transformation tend to cluster (apart) in the phylogenetic tree of the concatenate. This suggests that even though many T4aP might facilitate transformation, those effectively involved in transformation have evolved certain traits improving this function. One such feature is the presence of two disulphide bonds in pilins, which may stabilise the structure and improve retraction-force resistance of *Acinetobacter* [90] and enterobacterial T4aP major pilins [66] or of the competence-specific minor pilins in *Neisseria* [91].

Our study has revealed how a small set of proteins with different functions evolved to produce different adaptive functions involved in different types of motility, adherence, DNA uptake, and protein secretion. This process involved 1) accretion of accessory proteins, such as the secretin and secretin-associated proteins, to cope with the existence of an outer membrane in diderms; 2) duplication and subfunctionalisation of key components, such as pilins and ATPases; 3) internal gene duplication in the IM platform, followed by gene fission in TadBC; 4) several cases of gene loss, notably for some of the IM platform homologues in Tad, for the PilT ATPase, and for the secretin in monoderms; 5) gene transfer between distant clades, including a rare example of a large macromolecular system (Tad) transferred from Archaea to Bacteria; and 6) these events being accompanied by rearrangements of the genetic loci. TFFs were frequently transferred horizontally, which certainly accelerated their evolution because genetic exchanges break clonal interference and accelerate innovation processes by recombination [92]. Interestingly, we observed that genetic organisation and horizontal transfer were intimately associated, with systems encoded in one single locus showing higher rates of transfer. This may be a general pattern in the molecular evolution of complex systems in Bacteria and Archaea. Strong genetic linkage facilitates positive selection in physically interacting proteins [93] and the spread of the system to other species [63]. Novel genetic contexts may, in turn, select for further changes in the systems. Once functions remain for a long period of time in the lineage, as seems to be the case for ComM and some T4aP, major adaptive changes in the systems may become rare, and rearrangements splitting the initial locus may be eventually fixed. Radical changes in systems encoded in split loci are less likely to be spread by horizontal transfer, unless the recipient cell has already a copy of the system with similar genetic organisation. As a result, a tight association is established between genetic organisation and the ability of a system to evolve and spread by the action of horizontal gene transfer.

## Methods

### Data

We analysed 5,768 complete bacterial and archaeal genomes from NCBI RefSeq (ftp://ftp.ncbi.nlm.nih.gov/genomes/refseq/, last accessed in November 2016), representing 2,268 species of Bacteria and 168 species of Archaea.

### Detection of the TFF superfamily

All the systems of the family were detected using MacSyFinder v1.0.2 [44]. This program uses a model to identify a type of system in a DNA sequence (typically a replicon). The model specifies the components of the system, each represented by an HMM profile, and how their systems are organised in the sequence. A full description of the program and the models can be found in http://macsyfinder.readthedocs.io/en/latest/index.html. Briefly, the components can be mandatory, accessory, or forbidden. This does not represent a biological classification. The classification is made to distinguish between components that are ubiquitous and easy to identify (mandatory) and those that are either frequently absent or easily missed (accessory). A system is only validated if it fulfils a quorum of mandatory (minimum mandatory genes required [MMGR]) and/or mandatory + accessory genes (minimum genes required [MGR]). A locus is excluded if it contains a forbidden gene (these are useful to discriminate between closely related systems with a few specific components). Components are expected to be clustered in the genome at a short distance (defined in the model). Yet, some components can be defined as ‘loners’ and encoded apart. A component can be set as ‘exchangeable’, in which case several HMM profiles can be used to detect it (e.g., the same prepilin peptidase is used by T2SS and T4aP in some cases [49–51], and both profiles can be used to identify the prepilin peptidase of each of the two types of systems).

For this work, we could use the models previously proposed by TXSScan [39] for T2SS, T4aP, and Tad, but we wished to add a few components that were missing there. For the Archaeal-T4P and for ComM, we did not have an initial model. We proceeded in two steps. First, we made conservative initial models that matched the archetypal systems but sometimes were too strict for some atypical systems. This resulted in a list of systems in which we had strong confidence. However, it also missed many systems. To identify these systems, we built a model called ‘generic’ that had only the basic building blocks of these systems, with all the homologous proteins set as ‘exchangeable’. Following the comparative and phylogenetic analyses, we redefined all the models to make less-initial models that could identify a larger number of systems. Both sets of models are made available. The table with all protein profiles is given in S4 Table.

#### Generic

We defined the model called ‘generic’ to search for variants of the TFF superfamily that include the key components but do not fit the strict definitions of the more specific models (T4aP,T2SS, etc.). This model assumes that all the HMM profiles of the same connected component in the profile–profile graph of similarity can fill in for the function. Hence, it can identify very divergent or minimalistic systems, as well as chimeric systems with components that match profiles from different types. A cluster of components is classed as generic if it does not fit any of the more specific models and contains an ATPase, an IM platform, and a major pilin. In addition to these three proteins, the generic model also includes the prepilin peptidase and the secretin that are not deemed essential for the system because the former may be recruited from other systems in the genome [51], and the latter is specific to diderms.

#### Tad

The initial model of Tad closely followed the definitions proposed in [39]. This model includes all the known key components of the system and assumes that they are all encoded together, with the exception of *tadV*, the prepilin peptidase gene that can be encoded apart (loner) and be exchangeable with a number of homologous components from the T4aP (*pilD*) and ComM (*comC*).

The final model includes *tadD*, *rcpB*, and *rcpC* as new accessory components. The prepilin peptidase TadV is no longer exchangeable. The model defines the Tad pilus as multi_loci to allow for the existence of systems encoded in loci scattered in the genome (even if this is very rare).

#### T4aP

The initial model of T4aP was significantly improved from the model in [39]. It is more precise in the annotation of the retraction ATPases (*pilT* and *pilU*) and the major (*pilA*) and minor pilins (*pilE*, *pilX*, and *fimT*), and now accounting for five further components: *pilT*, *pilE*, *pilA*, and *fimT* set as mandatory and *pilU* and *pilX* set as accessory, according to their occurrence in the systems. Accordingly, the number of MGR and MMGR was increased to 8. The prepilin peptidase *pilD* was changed to mandatory, loner, and exchangeable with a number of homologous components from T2SS (*gspO*) and ComM (*comC*) according to its localisation, which could be found alone in the genome, and the fact that the HMM profiles of these two genes often have better e-value than the one of the T4aP.

The final model of T4aP includes *pilW*, *pilX*, and *pilY* as new accessory components. We decreased MMGR to 4 and MGR to 5, which better fit the data. We set *fimT*, *pilM*, *pilP*, and *pilA* as accessory to help MacSyFinder to search more complete T4aP in the genome. We also removed the forbidden genes *gspN*, *tadZ*, and *gspC*.

#### T2SS

The initial model of T2SS followed closely the definitions proposed in [39], in which we increased the MGR to 8 and set the prepilin peptidase *gspO* as mandatory, loner, and exchangeable with a number of homologous components from the T4aP (*pilD*).

The final model was relaxed to identify a larger fraction of the systems. We reduced the MMGR to 4 and the MGR to 5. To fit the data better, we added the prepilin peptidase of ComM (*comC*) as another exchangeable gene of *gspO*. We set the *gspC* gene as mandatory and *gspM* as accessory, and *gspD* was set as a loner to better fit the data.

#### ComM

In this initial model, only the genes that compose the pilus were used in the model, not the genes that encode DNA uptake system, such as *comEA*, *comEB*, and *comEC* [31,78,94–96]. The minimal distance between genes was set to 5. The MMGR was set to 3 and the MGR to 5, and the system was set as multi_loci because some genes are loners. The genes *comC*, *comGA*, *comGB*, *comGC*, and *comGD* were set as mandatory, and the other ones were set as accessory in relation with their presence in experimentally validated systems, curated with an exploratory phase to know the relative abundance of the genes in the systems (the genes with more than 80% of presence in the detected systems were set as mandatory and the others as accessory). *comC* was set as a loner and exchangeable with *pilD* of the T4aP because we found a case in which the HMM profile of *pilD* was better in e-value than that of *comC*, and for the same reason, *comGA* was set as exchangeable with *pilB* of T4aP. The genes *comB*, *comK*, and *comX* were set as loners because they are often found alone in the genome.

In the final model, we changed the number of genes for the MMGR and MGR to 4. We also added the genes encoding the DNA uptake system in the plasma membrane (*comEC*, *comEB*, and *comEA*). *comEC* was set as mandatory, *comEB* and *comEA* were set as accessory, and *comEC* and *comEB* were set as loners. Changing the gene *comGD* to accessory allowed us to search for loner genes without changing the MGR number.

#### Archaeal

Initial model. We here describe the first tool, to our knowledge, to detect Archaeal-T4P. We extracted the sequences from the 200 arCOG families (2014 version, [97]) deemed to be associated with Archaeal-T4P by Makarova and colleagues [35]. We built HMM profiles for each of these families: sequences were aligned with MAFFT v7.273 and linsi algorithm, and the alignment extremities were trimmed based on the results of BMGE with BLOSUM40 matrix [98,99]. HMM profiles were generated using HMMER version 3.1b2 [100]. These profiles were compared to profiles of Tad, T4aP, and T2SS from TXSScan [39] using HHsearch (e-value and *p*-value threshold of 0.001 for the family cutoff) in order to define suprafamilies of components [46]. Core ‘mandatory’ components were defined based on the literature and experimentally validated systems. Other components were set as ‘accessory’. The arCOG families that matched on the same component were defined as exchangeable. The prepilin peptidase was set as a loner gene that can be part of multiple systems. This initial model asked for a minimal number of mandatory genes and overall number of genes of 4. Of 14 experimentally validated systems found in the literature, 10 were detected with this initial model (S1 Table). After counting the occurrence of the different arCOGs in the detected Archaeal-T4P, we removed those without any occurrence to reduce the number to 109 arCOG families. Final model. The number of genes for MMGR and MGR was reduced to 3, which better fits the data.

#### T4bP

Final Model. This class includes the R64 thin pilus, toxin-coregulated pilus, bundle-forming pilus, longus pilus, and Cof pilus [27]. Because we do not have many experimentally validated systems for the T4bP, we used the phylogenetic information of the TFF superfamily trees to have a set of T4bP-related proteins to create the HMM profiles and the definition of the model. We created 8 HMM profiles; the MMGR was set to 4 and the MGR to 4. The system was set as multi_loci because some genes are loners. The genes *pilD*, *pilB*, *pilA*, *pilC*, and *pilQ* were set as mandatory, and the other ones were set as accessory, according to their occurrence in the detected systems. The prepilin peptidase *pilD* was set as a loner. *pilA* was set as exchangeable with *pilA* of T4aP because we found cases in which the HMM profile of *pilA* of T4aP had a better e-value in matches to T4bP than the *pilA* of T4bP.

#### MSH

Final Model. Because we do not have many experimentally validated systems for the MSH, we used the phylogenetic information of the TFF superfamily tree to have a set of MSH-related proteins to create the HMM profiles and the definition of the model. We created 20 HMM profiles; the MMGR was set to 3 and the MGR to 4. The system was set as multi_loci because some genes are loners. The genes *mshA*, *mshE*, *mshG*, *mshL*, and *mshM* were set as mandatory, and the others were set as accessory, according to their occurrence in the detected systems. The gene *mshA* was set as a loner, according to detected systems found in the genomes. *mshB* was set as exchangeable with *pilA* of T4bP because we found cases in which the HMM profile of *pilA* of T4bP provided better e-values when matching MSH systems than the *mshB* of MSH. For similar reasons, *mshC* was set as exchangeable with *fimT* of T4aP. The model for MSH does not include a prepilin peptidase because such a gene could not be identified in the locus.

### Retrieval and construction of protein profiles

We retrieved 37 profiles for T2SS, T4aP, and Tad from TXSScan [39]. For two HMM profiles of T4aP that combine the detection of two protein paralogues (T4P_pilT_pilU and T4P_pilAE), we decided to separate the sequence of the different proteins from the original alignment of this profile to generate five separate HMM profiles (T4P_pilT, T4P_pilU, T4P_pilA, T4P_fimT, and T4P_pilE).

To create the HMM profiles, we used the following methodology. For the genes that had few representatives in the experimentally validated data set, we used BLASTP v 2.5.0+ (default settings, e-value <1 × 10^−6^) [101] to search for homologues among complete genomes. To remove very closely related proteins, we performed an all-against-all BLASTP v2.5.0+ analysis and clustered the proteins with at least 80% sequence similarity using SiLiX v1.2.10-p1 (default settings) [102]. We selected one sequence from each family as a representative. We aligned all the representatives using MAFFT v7.273 (—auto, automatic selection of the parameters depending of the size of the alignment, default values for the other parameters) [98]. With SEAVIEW [103], the poorly aligned regions at the extremities were manually trimmed in the alignment. The trimmed alignment was used to build the HMM profile using hmmbuild (default parameters) from HMMER package v3.1b2 [104].

For the HMM profiles of the final model, we used the sequences of the profiles described above. Using the information of the phylogeny of the systems, we added the sequences of the systems that were annotated as generic but that clustered in a group of experimentally validated systems. We aligned all the representatives using MAFFT v7.273 (—auto, automatic selection of the parameters depending of the size of the alignment, default values for the other parameters) [98]. With SEAVIEW [103], the poorly aligned regions at the extremities were manually trimmed in the alignment. The trimmed alignment was used to build the HMM profile using hmmbuild (default parameters) from HMMER package v3.1b2 [104].

### Phylogenetic inference

Phylogenetic analyses based on protein sequences involved an initial alignment of the sequences using MAFFT v7.273 (linsi algorithm) [98]. Multiple alignments were analysed using Noisy v1.5.12 (default parameters) [105] to select the informative sites. We inferred maximum likelihood trees from the curated alignments or their concatenates, using IQ-TREE v 1.6.7.2 [52] (options -allnni, -nstop 1,000, -nm 100,000). We evaluated the node supports using the options -bb 1,000 for ultrafast bootstraps and -alrt 1,000 for SH-aLRT [106]. The best evolutionary model was selected with ModelFinder (option -MF, BIC criterion) [107]. We used the option -wbtl to conserve all optimal trees and their branch lengths.

The phylogenetic trees of 16S rRNA sequences were built from a data set including one sequence per genome of 5,776 genomes. The 16S sequences were retrieved from genome sequences using RNammer v1.2 [108] (options -S set to bac and the -m to ssu). We aligned archaeal and bacterial 16S rRNA separately using the secondary structure models with SSU_Align v0.1.1 (http://eddylab.org/software/ssu-align/, default options). Poorly aligned positions were masked with ssu-mask. The alignment was trimmed with trimAl v1.4rev15 [109] (-noallgaps, which allows for removing regions that are only composed of gaps from the alignment). The maximum likelihood trees were inferred using IQ-TREE v1.6.7.2 [52] (using the best-selected model SYM + R6 for the archaeal tree and SYM + R10 for the bacterial tree, -bb 10,000 ultrafast bootstrap [106], -wbtl to conserve all optimal trees and their branch lengths).

### Reference systems data set

The data set with all the systems identified in the genomes is too large to make phylogenetic inferences. It also contains many very closely related systems that may provide little additional information to infer the deeper nodes of the tree. Hence, we developed a method to remove redundancy in the data set while maximising its genetic diversity. The method prioritises the inclusion of systems that were experimentally validated to facilitate the analysis of the results. The method consists of several sequential steps (S15 Fig).

1. We inferred the maximum likelihood tree for each key components’ family as mentioned above and extracted the matrices of patristic distances (using the R function ‘cophenetic_phylo’ of the package ape) between all leaves of the trees. This resulted in a set of distance matrices between systems.
2. When there were multiple copies of a family of clade-specific paralogues, the system was represented multiple times in the phylogeny and in the distance matrix. To solve the problem and to have only one distance between two systems, we chose the minimal distance between the paralogues of the systems.
3. Each core protein family has a different rate of evolution. To compare them, we normalised each matrix by the sum of all the branch lengths in the tree of the family. We then built a matrix that is the average of all normalised matrices. This average matrix was used to infer a tree with bioNJ [110]. The tree was rooted at the midpoint.
4. We used the bioNJ tree to define monophyletic groups of similar systems. We iteratively used the R function ‘cutree’ from the stats package by gradually decreasing or increasing the heights at which the tree should be ‘cut’ until we obtained between 200 and 300 groups.
5. At this stage in the method, we had obtained a set of monophyletic groups of closely related systems. To pick the representative system of each group, we had the following order of priorities: i) inclusion of systems validated experimentally, ii) inclusion of the systems with fewest paralogues. In some rare cases, a given type of systems (e.g., T2SS) had less than 20 instances after this procedure. In this case, and to increase the statistical power of the analyses, we modified the height of the ‘cutree’ function for the specific subtree of the systems lacking representative to obtain a minimum of 20 systems for each group if possible. The systems selected to make the phylogeny are named ‘representative systems’.
6. We removed some complex systems from the reference ones (6/39 Archaeal-T4P and 10/101 generic) because they had two paralogues of all the genes or were generic systems with components from different types (e.g., T2SS_gspE and T4P_pilB).

### Dereplicated data set

To reduce the number of paralogues in each system, we used the following method (S16 Fig).

1. We inferred the maximum likelihood tree for each key components’ family of the representative data set as mentioned above and extracted the matrices of patristic distances (using the R function ‘cophenetic_phylo’ of the package ape) between all leaves of the trees. This resulted in a set of distance matrices between proteins.
2. For each system with more than one copy per gene, we found the nearest system, based on patristic distances extracted from the ATPase or the IM platform tree (depending on the number of copies of the ATPase), that had only one copy of this gene.
3. We use this nearest system to choose the copy of the duplicate gene with the smallest distance to its homologue in this nearest system.
4. In the end, each system is represented by a single instance of each of the core proteins, and we called this set of selected sequences and systems the ‘dereplicated data set’.

### Concatenate trees and ML topology tests

The Tad pilus and T4aP show cases of system-wide duplications: some of their gene families have several members in the same systems. That is the case of the IM platform for the Tad pilus (TadB and TadC are homologues that resulted from an initial gene internal duplication before the last common ancestor of TFFs, followed by a gene fission event) and for the ATPase of the T4aP (PilB and PilT/PilU are paralogues). Candidate systems’ trees were generated based on the concatenation of all possible combinations of putative sets of orthologues, i.e., each paralogue was picked one after the other to represent their system in phylogenetic analyses. E.g., for the IM platform, a first set of orthologues would consist of TadB sequences for Tad systems together with IM platform sequences from all other systems, and another would consist of TadC sequences for Tad systems together with IM platform sequences from all other systems. For the ATPase, we decided to only focus on the functional orthologous gene, the PilB sequences for T4aP systems.

Therefore, there were two possible combinations of the mandatory genes to generate concatenates of the ATPase IM platform. In total, we generated two concatenates and used IQ-Tree to compute maximum likelihood phylogenetic trees, using partition models (option -spp, the location of the genes in the concatenation defines the partitions, the model for each partition corresponds to the model found previously for the individual analyses).

In order to assess the congruence between the concatenate trees and the individual protein trees, a maximum likelihood topology test (AU for ‘Approximately Unbiased’ [111]) was performed using IQ-Tree. Each protein alignment was used as an input to assess the congruence of its ML tree with those of the 10 concatenate trees. The parameters of the model were estimated on the initial parsimony tree (option -n 0). A correction for multiple tests was applied to the *p*-values (sequential Bonferroni per batch of 10 concatenate trees).

### Analysis of the neighbourhood of the systems

We searched for genes systematically associated with a given type of systems by analysing the neighbourhood of each system. For each locus of a system, we identified its first gene (position X_First_) and last gene (position X_End_). We then took all the genes in a neighbourhood of 10 (i.e., between X_First-10_ and X_End+10_). When a system was encoded in multiple loci in the genome, each locus was analysed in the same way. We then clustered all these proteins by sequence similarity using BLASTP v. 2.5.0+ (default settings, e-value < 1 × 10^−6^) [101] and SiLiX v1.2.10-p1 (minimal percentage of identity to accept blast hits for building families at 50%) [102]. We kept the clusters if they had proteins represented in systems of different leaves in the tree. The proteins of each cluster were aligned with MAFFT and used to build HMM protein profiles as described above.

To annotate the protein clusters, we used two methods. First, we searched for similarities of their HMM profiles with the profiles used to identify the TFFs' components using HHsearch (v3.0.3, *p*-value < 1 × 10^−5^). Second, we searched for homologies between the remaining clusters and the profiles of the PFAM database (v31.0, same method).

To test whether a given cluster is significantly positively associated with a given type of system, we made the following analysis. We counted the occurrences of the elements of the cluster associated with a given type and made a contingency table in which the columns are the type versus all other types and the lines are presence or absence of an element from the cluster. To test statistical significance, we used a Fisher's exact test on the contingency table. Because this implicates many statistical tests—one test per type per cluster and this for many clusters and several types—we adjusted the *p*-values for multiple tests using the Bonferroni correction. We kept the association between a given cluster and a given type of system if the number of elements in the cluster neighbouring systems of that type was higher than expected by chance and if the corrected *p*-value < 0.05. The resulting matrix of presence/absence for genes positively associated with the systems can be found in S5 Table.

### Inferring transfers of systems

We took the phylogenetic tree of all systems and picked the subtrees of each type of system. For each of these trees, we pruned the 16S rRNA tree such that it only includes species present in the system tree. We used ALE v0.4 (default parameters), a reconciliation program that introduces events of duplication, transfer, and loss (DTL) in a gene tree, and amalgamated the most frequent subtrees in a sample distribution of the gene tree to improve it and make it congruent with the species (reference) tree in a maximum likelihood framework [112]. Using ALE, we computed a number of DTL events introduced in each system’s trees given the 16S rRNA tree as a reference. We then computed the proportion of transfers by collecting the number of transfers for each type of system and dividing it by the number of branches in the subtrees of each type of system.

### Analysis of genetic organisation

We identified all pairs of contiguous components of the systems (for a gene X_p_ in the cluster, we look at the genes X_p − 1_ and X_p + 1_). We constructed an adjacency matrix using this information, and we used it to construct a graph of the genetic organisation of the systems. We normalised the association between two genes to represent two different types of information:

1. To know how frequently two genes are contiguous, we divided the number of contiguous occurrences by the number of occurrences of the rarest of the two genes. This corresponds to the edge widths in Fig 5.
2. To know how many times the contiguity is found in the system, we divided the number of times the contiguity is observed by the number of systems detected. This corresponds to the edge colours in Fig 5.

## Supporting information

S1 Fig Representation of the initial and final models of the systems. Homologous genes are indicated by the same colour. Mandatory genes are indicated with a full outline, accessory genes are indicated with a dash outline, and forbidden genes are indicated with a red cross. The exchangeable genes are indicated by an arrow. The loner genes are indicated by a star below the gene. For the Archaeal-T4P, the aCXXX name indicates that all the homologous arCOGs for this function (they are set as exchangeable). The empty box in the genetic model indicates that the genes are exchangeable with all the homologous genes of the other models. Archaeal-T4P, type IV-related pili in Archaea; arCOG, archaeal Cluster of Orthologous Genes.
(PDF)

S2 Fig Taxonomic distribution of the systems in Bacteria and Archaea with the initial models. Cells indicate the number of genomes with at least one detected system. The cell’s colour gradient represents the proportion of genomes with at least one system in the clade. The bar plot shows the total number of detected systems. The bars are separated in two categories: Alpha-, Beta-, and Gamma-proteobacteria versus the other clades. The cladogram symbolises approximated relationships between the bacterial and archaeal taxa analysed in this study.
(PDF)

S3 Fig Rooted phylogeny of the ATPase. The colour of the label of the leaves indicates the taxonomic group of the species. The different coloured strips indicate the classification of the systems with the MacSyFinder annotation (with the initial model and with the final one) and the annotation of the systems in the literature. The systems known to be implicated in natural transformation are indicated in dark purple. Known subtypes of Archaeal-T4P are indicate by text in red. The annotation of the domains of the proteins using are also added. The tree was built using IQ-Tree, 10,000 replicates of UFBoot, model LG + 10. Halo pilus indicates two pili characterised in Halobacteria. Archaeal-T4P, type IV-related pili in Archaea; UFBoot, Ultrafast Bootstrap Approximation.
(PDF)

S4 Fig Rooted phylogeny of the ATPase with FtsK as external group. The colour of the label of the leaves indicates the taxonomic group of the species. The different coloured strips indicate the classification of the systems with the MacSyFinder annotation (with the initial model and with the final one) and the annotation of the systems in the literature. The systems known to be implicated in natural transformation are indicated in dark purple. Known subtypes of Archaeal-T4P are indicate by text in red. The annotation of the domains of the proteins using are also added. The tree was built using IQ-Tree, 10,000 replicates of UFBoot, model LG + R10. Halo pilus indicates two pili characterised in Halobacteria. Archaeal-T4P, type IV-related pili in Archaea; UFBoot, Ultrafast Bootstrap Approximation.
(PDF)

S5 Fig Rooted phylogeny of the ATPase with FtsK and virB4 as external group. The colour of the label of the leaves indicates the taxonomic group of the species. The different coloured strips indicate the classification of the systems with the MacSyFinder annotation (with the initial model and with the final one) and the annotation of the systems in the literature. The systems known to be implicated in natural transformation are indicated in dark purple. Known subtypes of Archaeal-T4P are indicate by text in red. The annotation of the domains of the proteins using are also added. The tree was built using IQ-Tree, 10,000 replicates of UFBoot, model LG + R9. Halo pilus indicates two pili characterised in Halobacteria. Archaeal-T4P, type IV-related pili in Archaea; UFBoot, Ultrafast Bootstrap Approximation.
(PDF)

S6 Fig Rooted phylogeny of the IM platform. The colour of the label of the leaves indicates the taxonomic group of the species. The different coloured strips indicate the classification of the systems with the MacSyFinder annotation (with the initial model and with the final one) and the annotation of the systems in the literature. The systems known to be implicated in natural transformation are indicated in dark purple. Known subtypes of Archaeal-T4P are indicate by text in red. The annotation of the domains of the proteins using are also added. The tree was built using IQ-Tree, 10,000 replicates of UFBoot, model LG + F + R8. Halo pilus indicates two pili characterised in Halobacteria. Archaeal-T4P, type IV-related pili in Archaea; IM, integral membrane; UFBoot, Ultrafast Bootstrap Approximation.
(PDF)

S7 Fig Rooted phylogeny of the major pilin. The colour of the label of the leaves indicates the taxonomic group of the species. The different coloured strips indicate the classification of the systems with the MacSyFinder annotation (with the initial model and with the final one) and the annotation of the systems in the literature. The systems known to be implicated in natural transformation are indicated in dark purple. Known subtypes of Archaeal-T4P are indicate by text in red. The annotation of the domains of the proteins using are also added. The tree was built using IQ-Tree, 10,000 replicates of UFBoot, model LG + F + R7. Halo pilus indicates two pili characterised in Halobacteria. Archaeal-T4P, type IV-related pili in Archaea; UFBoot, Ultrafast Bootstrap Approximation.
(PDF)

S8 Fig Unrooted phylogeny of the prepilin peptidase. The colour of the label of the leaves indicates the taxonomic group of the species. The different coloured strips indicate the classification of the systems with the MacSyFinder annotation (with the initial model and with the final one) and the annotation of the systems in the literature. The systems known to be implicated in natural transformation are indicated in dark purple. Known subtypes of Archaeal-T4P are indicate by text in red. The annotation of the domains of the proteins used are also added. The tree was built using IQ-Tree, 10,000 replicates of UFBoot, model VT + F + R6. Archaeal-T4P, type IV-related pili in Archaea; UFBoot, Ultrafast Bootstrap Approximation.
(PDF)

S9 Fig Unrooted phylogeny of the secretin. The colour of the label of the leaves indicates the taxonomic group of the species. The different coloured strips indicate the classification of the systems with the MacSyFinder annotation (with the initial model and with the final one) and the annotation of the systems in the literature. The systems known to be implicated in natural transformation are indicated in dark purple. Known subtypes of Archaeal-T4P are indicate by text in red. The annotation of the domains of the proteins used are also added. The tree was built using IQ-Tree, 10,000 replicates of UFBoot, model LG + F + R8. Archaeal-T4P, type IV-related pili in Archaea; UFBoot, Ultrafast Bootstrap Approximation.
(PDF)

S10 Fig Rooted phylogeny of the TFF superfamily. The tree was built with the concatenate of the IM platform (using TadB) and the AAA+ ATPase (using PilB). The colour of the label of the leaves indicates the taxonomic group of the species. The different coloured strips indicate the classification of the systems with the MacSyFinder annotation (with the initial model and with the final one) and the annotation of the systems in the literature. The systems known to be implicated in natural transformation are indicated in dark purple. Known subtypes of Archaeal-T4P are indicate by text in red. The tree was built using IQ-Tree, 10,000 replicates of UFBoot, with a partition model. Halo pilus indicates two pili characterised in Halobacteria. Archaeal-T4P, type IV-related pili in Archaea; Tad, tight adherence; TFF, type IV filament; UF, Ultrafast Bootstrap Approximation.
(PDF)

S11 Fig Taxonomic distribution of the systems in Bacteria and Archaea using the phylogenetic clustering to annotate generic systems. Cells indicate the number of genomes with at least one detected system. The cell’s colour gradient represents the proportion of genomes with at least one system in the clade. The bar plot shows the total number of detected systems. The bars are separated into two categories: Alpha-, Beta-, and Gamma-proteobacteria versus the other clades. The cladogram symbolises approximated relationships between the bacterial and archaeal taxa analysed in this study.
(PDF)

S12 Fig Genetic organisation of the Archaeal-T4P in genomes. The edge width represents the number of times the two genes are contiguous divided by the number of times the rarest gene is present in the system. The colour of the edge represents the number of times the two genes are contiguous in the system divided by the number of systems. Archaeal-T4P, type IV-related pili in Archaea.
(PDF)

S13 Fig Genetic organisation of the archaellum in genomes. The edge width represents the number of times the two genes are contiguous divided by the number of times the rarest gene is present in the system. The colour of the edge represents the number of times the two genes are contiguous in the system divided by the number of systems.
(PDF)

S14 Fig 16S tree used to infer horizontal transfers. The colour of the leaves represents the phyla of the Bacteria. The tree was built using IQ-Tree, 1,000 replicates of UFBoot, model SYM + R10. UFBoot, Ultrafast Bootstrap Approximation.
(PDF)

S15 Fig Schema of the workflow used to choose the representative systems. (PDF)

S16 Fig Schema of the workflow used to choose the species-specific paralogues. (PDF)

S1 Table List of all the profiles of the TFF superfamily used in the analysis. TFF, type IV filament.
(XLSX)

S2 Table Experimentally validated systems used in the analysis. (XLSX)

S3 Table Description of all the genes and concatenate trees inferred in this study. (XLSX)

S4 Table All trees inferred in this study in newick format. (TXT)

S5 Table Matrix of presence/absence of neighbouring genes positively associated with the systems (family of genes is in columns and systems are in rows). (XLSX)

S6 Table All the systems detected by MacSyFinder with the search using the final models. (XLSX)

S7 Table Tree topology tests (AU) using IQ-TREE between concatenated trees and the genes that compose the concatenate. AU, Approximately Unbiased.
(XLSX)

S8 Table Global scenario of TFF evolution: Summary of the evolutionary events presented in Fig 7. TFF, type IV filament.
(PDF)

## Abbreviations

Aap: Archaeal adhesive pilus
ABC: ATP-binding cassette
Archaeal-T4P: type IV-related pili in Archaea
arCOG: archaeal Cluster of Orthologous Genes
AU: Approximately Unbiased
Bas: Bindosome
Ced: Crenarchaeal system for exchange of DNA
Com: competence pilus
ComM: Com of monoderms
DTL: duplication, transfer, or loss
Epd: EppA dependent
HMM: hidden Markov model
IM: integral membrane
MGR: minimum genes required
MMGR: minimum mandatory genes required
MSH: mannose-sensitive hemagglutinin pilus
SDA: secretin-dynamic–associated
Tad: tight adherence
TFF: type IV filament
T2SS: type II protein secretion system
T3SS: type III protein secretion system
T4aP: type IVa pilus
T4bP: type IVb pilus
Ups: UV-inducible pilus of *Sulfolobus*
UFBoot: Ultrafast Bootstrap Approximation

## References

[1] Gould SJ, Vrba ES. Exaptation—a Missing Term in the Science of Form. Paleobiology. 1982;8(1):4–15.

[2] Pal C, Papp B, Lercher MJ. An integrated view of protein evolution. Nat Rev Genet. 2006;7:337–48. 10.1038/nrg1838 16619049

[3] Jacob F. Evolution and tinkering. Science. 1977;196(4295):1161–6. 10.1126/science.860134 860134

[4] Ochman H, Lawrence JG, Groisman EA. Lateral gene transfer and the nature of bacterial innovation. Nature. 2000;405(6784):299–304. 10.1038/35012500 10830951

[5] Gogarten JP, Townsend JP. Horizontal gene transfer, genome innovation and evolution. Nat Rev Microbiol. 2005;3(9):679–87. 10.1038/nrmicro1204 16138096

[6] Jain R, Rivera MC, Moore JE, Lake JA. Horizontal gene transfer accelerates genome innovation and evolution. Mol Biol Evol. 2003;20(10):1598–602. 10.1093/molbev/msg154 12777514

[7] Szappanos B, Fritzemeier J, Csorgo B, Lazar V, Lu X, Fekete G, et al Adaptive evolution of complex innovations through stepwise metabolic niche expansion. Nat Commun. 2016;7:11607 10.1038/ncomms11607 27197754 PMC5411730

[8] Abby SS, Rocha EP. The non-flagellar type III secretion system evolved from the bacterial flagellum and diversified into host-cell adapted systems. PLoS Genet. 2012;8(9):e1002983 10.1371/journal.pgen.1002983 23028376 PMC3459982

[9] Guglielmini J, de la Cruz F, Rocha EP. Evolution of conjugation and type IV secretion systems. Mol Biol Evol. 2013;30(2):315–31. 10.1093/molbev/mss221 22977114 PMC3548315

[10] Pell LG, Kanelis V, Donaldson LW, Howell PL, Davidson AR. The phage lambda major tail protein structure reveals a common evolution for long-tailed phages and the type VI bacterial secretion system. Proc Natl Acad Sci U S A. 2009;106(11):4160–5. 10.1073/pnas.0900044106 19251647 PMC2657425

[11] Leiman PG, Basler M, Ramagopal UA, Bonanno JB, Sauder JM, Pukatzki S, et al Type VI secretion apparatus and phage tail-associated protein complexes share a common evolutionary origin. Proc Natl Acad Sci U S A. 2009;106(11):4154–9. 10.1073/pnas.0813360106 19251641 PMC2657435

[12] Peabody CR, Chung YJ, Yen MR, Vidal-Ingigliardi D, Pugsley AP, Saier MH Jr. Type II protein secretion and its relationship to bacterial type IV pili and archaeal flagella. Microbiology. 2003;149(Pt 11):3051–72. 10.1099/mic.0.26364-0 14600218

[13] Jarrell KF, Albers SV. The archaellum: an old motility structure with a new name. Trends Microbiol. 2012;20(7):307–12. 10.1016/j.tim.2012.04.007 22613456

[14] Berry JL, Pelicic V. Exceptionally widespread nanomachines composed of type IV pilins: the prokaryotic Swiss Army knives. FEMS Microbiol Rev. 2015;39(1):134–54. 10.1093/femsre/fuu001 25793961 PMC4471445

[15] Gold V, Kudryashev M. Recent progress in structure and dynamics of dual-membrane-spanning bacterial nanomachines. Curr Opin Struct Biol. 2016;39:1–7. 10.1016/j.sbi.2016.03.001 26995496

[16] Korotkov KV, Sandkvist M, Hol WG. The type II secretion system: biogenesis, molecular architecture and mechanism. Nat Rev Microbiol. 2012;10(5):336–51. 10.1038/nrmicro2762 22466878 PMC3705712

[17] Korotkov KV, Gonen T, Hol WG. Secretins: dynamic channels for protein transport across membranes. Trends Biochem Sci. 2011;36(8):433–43. 10.1016/j.tibs.2011.04.002 21565514 PMC3155655

[18] Jouravleva EA, McDonald GA, Marsh JW, Taylor RK, Boesman-Finkelstein M, Finkelstein RA. The Vibrio cholerae mannose-sensitive hemagglutinin is the receptor for a filamentous bacteriophage from V. cholerae O139. Infect Immun. 1998;66(6):2535–9. 9596713 10.1128/iai.66.6.2535-2539.1998 PMC108235

[19] Marsh JW, Taylor RK. Genetic and transcriptional analyses of the Vibrio cholerae mannose-sensitive hemagglutinin type 4 pilus gene locus. J Bacteriol. 1999;181(4):1110–7. 9973335 10.1128/jb.181.4.1110-1117.1999 PMC93486

[20] Fitzgerald LA, Petersen ER, Ray RI, Little BJ, Cooper CJ, Howard EC, et al Shewanella oneidensis MR-1 Msh pilin proteins are involved in extracellular electron transfer in microbial fuel cells. Process Biochemistry. 2012;47(1):170–4.

[21] Rakhuba DV, Kolomiets EI, Dey ES, Novik GI. Bacteriophage receptors, mechanisms of phage adsorption and penetration into host cell. Pol J Microbiol. 2010;59(3):145–55. 21033576

[22] Wairuri CK, van der Waals JE, van Schalkwyk A, Theron J. Ralstonia solanacearum needs Flp pili for virulence on potato. Mol Plant Microbe Interact. 2012;25(4):546–56. 10.1094/MPMI-06-11-0166 22168446

[23] Hirst TR, Sanchez J, Kaper JB, Hardy SJ, Holmgren J. Mechanism of toxin secretion by Vibrio cholerae investigated in strains harboring plasmids that encode heat-labile enterotoxins of Escherichia coli. Proc Natl Acad Sci U S A. 1984;81(24):7752–6. 10.1073/pnas.81.24.7752 6393126 PMC392230

[24] Sikora AE, Zielke RA, Lawrence DA, Andrews PC, Sandkvist M. Proteomic analysis of the Vibrio cholerae type II secretome reveals new proteins, including three related serine proteases. J Biol Chem. 2011;286(19):16555–66. 10.1074/jbc.M110.211078 21385872 PMC3089498

[25] Cadoret F, Ball G, Douzi B, Voulhoux R. Txc, a new type II secretion system of Pseudomonas aeruginosa strain PA7, is regulated by the TtsS/TtsR two-component system and directs specific secretion of the CbpE chitin-binding protein. J Bacteriol. 2014;196(13):2376–86. 10.1128/JB.01563-14 24748613 PMC4054165

[26] DebRoy S, Dao J, Soderberg M, Rossier O, Cianciotto NP. Legionella pneumophila type II secretome reveals unique exoproteins and a chitinase that promotes bacterial persistence in the lung. Proc Natl Acad Sci U S A. 2006;103(50):19146–51. 10.1073/pnas.0608279103 17148602 PMC1748190

[27] Roux N, Spagnolo J, de Bentzmann S. Neglected but amazingly diverse type IVb pili. Res Microbiol. 2012;163(9–10):659–73. 10.1016/j.resmic.2012.10.015 23103334

[28] Mazariego-Espinosa K, Cruz A, Ledesma MA, Ochoa SA, Xicohtencatl-Cortes J. Longus, a type IV pilus of enterotoxigenic Escherichia coli, is involved in adherence to intestinal epithelial cells. J Bacteriol. 2010;192(11):2791–800. 10.1128/JB.01595-09 20348256 PMC2876479

[29] Skerker JM, Berg HC. Direct observation of extension and retraction of type IV pili. Proc Natl Acad Sci U S A. 2001;98(12):6901–4. 10.1073/pnas.121171698 11381130 PMC34450

[30] Wagner A, Whitaker RJ, Krause DJ, Heilers JH, van Wolferen M, van der Does C, et al Mechanisms of gene flow in archaea. Nat Rev Microbiol. 2017;15(8):492–501. 10.1038/nrmicro.2017.41 28502981

[31] Johnston C, Martin B, Fichant G, Polard P, Claverys JP. Bacterial transformation: distribution, shared mechanisms and divergent control. Nat Rev Microbiol. 2014;12(3):181–96. 10.1038/nrmicro3199 24509783

[32] Hofreuter D, Odenbreit S, Haas R. Natural transformation competence in Helicobacter pylori is mediated by the basic components of a type IV secretion system. Mol Microbiol. 2001;41(2):379–91. 11489125 10.1046/j.1365-2958.2001.02502.x

[33] Patenge N, Berendes A, Engelhardt H, Schuster SC, Oesterhelt D. The fla gene cluster is involved in the biogenesis of flagella in Halobacterium salinarum. Mol Microbiol. 2001;41(3):653–63. 11532133 10.1046/j.1365-2958.2001.02542.x

[34] Ng SY, Chaban B, Jarrell KF. Archaeal flagella, bacterial flagella and type IV pili: a comparison of genes and posttranslational modifications. J Mol Microbiol Biotechnol. 2006;11(3–5):167–91. 10.1159/000094053 16983194

[35] Makarova KS, Koonin EV, Albers SV. Diversity and Evolution of Type IV pili Systems in Archaea. Front Microbiol. 2016;7:667 10.3389/fmicb.2016.00667 27199977 PMC4858521

[36] Pelicic V. Type IV pili: e pluribus unum? Mol Microbiol. 2008;68(4):827–37. 10.1111/j.1365-2958.2008.06197.x 18399938

[37] Tomich M, Planet PJ, Figurski DH. The tad locus: postcards from the widespread colonization island. Nat Rev Microbiol. 2007;5(5):363–75. 10.1038/nrmicro1636 17435791

[38] Pugsley AP. The complete general secretory pathway in gram-negative bacteria. Microbiol Rev. 1993;57(1):50–108. 8096622 10.1128/mr.57.1.50-108.1993 PMC372901

[39] Abby SS, Cury J, Guglielmini J, Neron B, Touchon M, Rocha EP. Identification of protein secretion systems in bacterial genomes. Sci Rep. 2016;6:23080 10.1038/srep23080 26979785 PMC4793230

[40] Planet PJ, Kachlany SC, DeSalle R, Figurski DH. Phylogeny of genes for secretion NTPases: identification of the widespread tadA subfamily and development of a diagnostic key for gene classification. Proc Natl Acad Sci U S A. 2001;98(5):2503–8. 10.1073/pnas.051436598 11226268 PMC30167

[41] Iyer LM, Makarova KS, Koonin EV, Aravind L. Comparative genomics of the FtsK-HerA superfamily of pumping ATPases: implications for the origins of chromosome segregation, cell division and viral capsid packaging. Nucleic Acids Res. 2004;32(17):5260–79. 10.1093/nar/gkh828 15466593 PMC521647

[42] Desmond E, Brochier-Armanet C, Gribaldo S. Phylogenomics of the archaeal flagellum: rare horizontal gene transfer in a unique motility structure. BMC Evol Biol. 2007;7:106 10.1186/1471-2148-7-106 17605801 PMC1914349

[43] Ellison CK, Kan J, Dillard RS, Kysela DT, Ducret A, Berne C, et al Obstruction of pilus retraction stimulates bacterial surface sensing. Science. 2017;358(6362):535–8. 10.1126/science.aan5706 29074778 PMC5805138

[44] Abby SS, Neron B, Menager H, Touchon M, Rocha EP. MacSyFinder: a program to mine genomes for molecular systems with an application to CRISPR-Cas systems. PLoS ONE. 2014;9(10):e110726 10.1371/journal.pone.0110726 25330359 PMC4201578

[45] Abby SS, Rocha EPC. Identification of Protein Secretion Systems in Bacterial Genomes Using MacSyFinder. Methods Mol Biol. 2017;1615:1–21. 10.1007/978-1-4939-7033-9_1 28667599

[46] Soding J. Protein homology detection by HMM-HMM comparison. Bioinformatics. 2005;21(7):951–60. 10.1093/bioinformatics/bti125 15531603

[47] Xu Q, Christen B, Chiu HJ, Jaroszewski L, Klock HE, Knuth MW, et al Structure of the pilus assembly protein TadZ from Eubacterium rectale: implications for polar localization. Mol Microbiol. 2012;83(4):712–27. 10.1111/j.1365-2958.2011.07954.x 22211578 PMC3272108

[48] Perez-Cheeks BA, Planet PJ, Sarkar IN, Clock SA, Xu Q, Figurski DH. The product of tadZ, a new member of the parA/minD superfamily, localizes to a pole in Aggregatibacter actinomycetemcomitans. Mol Microbiol. 2012;83(4):694–711. 10.1111/j.1365-2958.2011.07955.x 22239271 PMC3305808

[49] Nunn DN, Lory S. Product of the Pseudomonas aeruginosa gene pilD is a prepilin leader peptidase. Proc Natl Acad Sci U S A. 1991;88(8):3281–5. 10.1073/pnas.88.8.3281 1901657 PMC51430

[50] Pepe CM, Eklund MW, Strom MS. Cloning of an Aeromonas hydrophila type IV pilus biogenesis gene cluster: complementation of pilus assembly functions and characterization of a type IV leader peptidase/N-methyltransferase required for extracellular protein secretion. Mol Microbiol. 1996;19(4):857–69. 8820654 10.1046/j.1365-2958.1996.431958.x

[51] Marsh JW, Taylor RK. Identification of the Vibrio cholerae type 4 prepilin peptidase required for cholera toxin secretion and pilus formation. Mol Microbiol. 1998;29(6):1481–92. 9781884 10.1046/j.1365-2958.1998.01031.x

[52] Nguyen LT, Schmidt HA, von Haeseler A, Minh BQ. IQ-TREE: a fast and effective stochastic algorithm for estimating maximum-likelihood phylogenies. Mol Biol Evol. 2015;32(1):268–74. 10.1093/molbev/msu300 25371430 PMC4271533

[53] Sherratt DJ, Arciszewska LK, Crozat E, Graham JE, Grainge I. The Escherichia coli DNA translocase FtsK. Biochem Soc Trans. 2010;38(2):395–8. 10.1042/BST0380395 20298190

[54] Nolivos S, Touzain F, Pages C, Coddeville M, Rousseau P, El Karoui M, et al Co-evolution of segregation guide DNA motifs and the FtsK translocase in bacteria: identification of the atypical Lactococcus lactis KOPS motif. Nucleic Acids Res. 2012;40(12):5535–45. 10.1093/nar/gks171 22373923 PMC3384302

[55] Iwabe N, Kuma K, Hasegawa M, Osawa S, Miyata T. Evolutionary relationship of archaebacteria, eubacteria, and eukaryotes inferred from phylogenetic trees of duplicated genes. Proc Natl Acad Sci U S A. 1989;86(23):9355–9. 10.1073/pnas.86.23.9355 2531898 PMC298494

[56] Gogarten JP, Kibak H, Dittrich P, Taiz L, Bowman EJ, Bowman BJ, et al Evolution of the vacuolar H+-ATPase: implications for the origin of eukaryotes. Proc Natl Acad Sci U S A. 1989;86(17):6661–5. 10.1073/pnas.86.17.6661 2528146 PMC297905

[57] Imam S, Chen Z, Roos DS, Pohlschroder M. Identification of surprisingly diverse type IV pili, across a broad range of gram-positive bacteria. PLoS ONE. 2011;6(12):e28919 10.1371/journal.pone.0028919 22216142 PMC3244431

[58] Wang X, Han Q, Chen G, Zhang W, Liu W. A Putative Type II Secretion System Is Involved in Cellulose Utilization in Cytophaga hutchisonii. Front Microbiol. 2017;8:1482 10.3389/fmicb.2017.01482 28848505 PMC5553014

[59] Zeng L, Zhang Y, Zhu Y, Yin H, Zhuang X, Zhu W, et al Extracellular proteome analysis of Leptospira interrogans serovar Lai. OMICS. 2013;17(10):527–35. 10.1089/omi.2013.0043 23895271 PMC3783971

[60] Nguyen BD, Valdivia RH. Virulence determinants in the obligate intracellular pathogen Chlamydia trachomatis revealed by forward genetic approaches. Proc Natl Acad Sci U S A. 2012;109(4):1263–8. 10.1073/pnas.1117884109 22232666 PMC3268281

[61] Achaz G, Rocha EP, Netter P, Coissac E. Origin and fate of repeats in bacteria. Nucleic Acids Res. 2002;30(13):2987–94. 10.1093/nar/gkf391 12087185 PMC117046

[62] Planet PJ, Kachlany SC, Fine DH, DeSalle R, Figurski DH. The Widespread Colonization Island of Actinobacillus actinomycetemcomitans. Nat Genet. 2003;34(2):193–8. 10.1038/ng1154 12717435

[63] Lawrence JG, Roth JR. Selfish operons: horizontal transfer may drive the evolution of gene clusters. Genetics. 1996;143(4):1843–60. 8844169 10.1093/genetics/143.4.1843 PMC1207444

[64] El-Gebali S, Mistry J, Bateman A, Eddy SR, Luciani A, Potter SC, et al The Pfam protein families database in 2019. Nucleic Acids Res. 2019;47(D1):D427–D32. 10.1093/nar/gky995 30357350 PMC6324024

[65] Iwata M, Imamura H, Stambouli E, Ikeda C, Tamakoshi M, Nagata K, et al Crystal structure of a central stalk subunit C and reversible association/dissociation of vacuole-type ATPase. Proc Natl Acad Sci U S A. 2004;101(1):59–64. 10.1073/pnas.0305165101 14684831 PMC314138

[66] Luna Rico A, Zheng W, Petiot N, Egelman EH, Francetic O. Functional reconstitution of the type IVa pilus assembly system from enterohaemorrhagic Escherichia coli. Mol Microbiol. 2019;111(3):732–749. 10.1111/mmi.14188 30561149 PMC6417937

[67] Nivaskumar M, Santos-Moreno J, Malosse C, Nadeau N, Chamot-Rooke J, Tran Van Nhieu G, et al Pseudopilin residue E5 is essential for recruitment by the type 2 secretion system assembly platform. Mol Microbiol. 2016;101(6):924–41. 10.1111/mmi.13432 27260845

[68] Goosens VJ, Busch A, Georgiadou M, Castagnini M, Forest KT, Waksman G, et al Reconstitution of a minimal machinery capable of assembling periplasmic type IV pili. Proc Natl Acad Sci U S A. 2017;114(25):E4978–E86. 10.1073/pnas.1618539114 28588140 PMC5488919

[69] Raymann K, Brochier-Armanet C, Gribaldo S. The two-domain tree of life is linked to a new root for the Archaea. Proc Natl Acad Sci U S A. 2015;112(21):6670–5. 10.1073/pnas.1420858112 25964353 PMC4450401

[70] O'Connell Motherway M, Zomer A, Leahy SC, Reunanen J, Bottacini F, Claesson MJ, et al Functional genome analysis of Bifidobacterium breve UCC2003 reveals type IVb tight adherence (Tad) pili as an essential and conserved host-colonization factor. Proc Natl Acad Sci U S A. 2011;108(27):11217–22. 10.1073/pnas.1105380108 21690406 PMC3131351

[71] Nair DB, Uchida K, Aizawa S, Jarrell KF. Genetic analysis of a type IV pili-like locus in the archaeon Methanococcus maripaludis. Arch Microbiol. 2014;196(3):179–91. 10.1007/s00203-014-0956-4 24493292

[72] Clock SA, Planet PJ, Perez BA, Figurski DH. Outer membrane components of the Tad (tight adherence) secreton of Aggregatibacter actinomycetemcomitans. J Bacteriol. 2008;190(3):980–90. 10.1128/JB.01347-07 18055598 PMC2223556

[73] Antunes LC, Poppleton D, Klingl A, Criscuolo A, Dupuy B, Brochier-Armanet C, et al Phylogenomic analysis supports the ancestral presence of LPS-outer membranes in the Firmicutes. Elife. 2016;5:e14589 10.7554/eLife.14589 27580370 PMC5007114

[74] Nickerson NN, Abby SS, Rocha EP, Chami M, Pugsley AP. A Single Amino Acid Substitution Changes the Self-Assembly Status of a Type IV Piliation Secretin. J Bacteriol. 2012;194(18):4951–8. 10.1128/JB.00798-12 22773793 PMC3430340

[75] Ellison CK, Kan J, Chlebek JL, Hummels KR, Panis G, Viollier PH, et al. A bifunctional ATPase drives tad pilus extension and retraction. bioRxiv. 616128 [Preprint] [cited 2019 Jun 3]. Available from: https://www.biorxiv.org/content/10.1101/616128v1. 10.1126/sciadv.aay2591 PMC6920026 31897429

[76] Ellison CK, Dalia TN, Vidal Ceballos A, Wang JC, Biais N, Brun YV, et al Retraction of DNA-bound type IV competence pili initiates DNA uptake during natural transformation in Vibrio cholerae. Nat Microbiol. 2018;3(7):773–80. 10.1038/s41564-018-0174-y 29891864 PMC6582970

[77] Seitz P, Blokesch M. DNA-uptake machinery of naturally competent Vibrio cholerae. Proc Natl Acad Sci U S A. 2013;110(44):17987–92. 10.1073/pnas.1315647110 24127573 PMC3816411

[78] Laurenceau R, Pehau-Arnaudet G, Baconnais S, Gault J, Malosse C, Dujeancourt A, et al A type IV pilus mediates DNA binding during natural transformation in Streptococcus pneumoniae. PLoS Pathog. 2013;9(6):e1003473 10.1371/journal.ppat.1003473 23825953 PMC3694846

[79] Carruthers MD, Tracy EN, Dickson AC, Ganser KB, Munson RS Jr., Bakaletz LO. Biological roles of nontypeable Haemophilus influenzae type IV pilus proteins encoded by the pil and com operons. J Bacteriol. 2012;194(8):1927–33. 10.1128/JB.06540-11 22328674 PMC3318474

[80] Pohlschroder M, Esquivel RN. Archaeal type IV pili and their involvement in biofilm formation. Front Microbiol. 2015;6:190 10.3389/fmicb.2015.00190 25852657 PMC4371748

[81] Tala L, Fineberg A, Kukura P, Persat A. Pseudomonas aeruginosa orchestrates twitching motility by sequential control of type IV pili movements. Nat Microbiol. 2019;4(5):774–80. 10.1038/s41564-019-0378-9 30804544 PMC6522360

[82] van Wolferen M, Wagner A, van der Does C, Albers SV. The archaeal Ced system imports DNA. Proc Natl Acad Sci U S A. 2016;113(9):2496–501. 10.1073/pnas.1513740113 26884154 PMC4780597

[83] Frols S, Ajon M, Wagner M, Teichmann D, Zolghadr B, Folea M, et al UV-inducible cellular aggregation of the hyperthermophilic archaeon Sulfolobus solfataricus is mediated by pili formation. Mol Microbiol. 2008;70(4):938–52. 10.1111/j.1365-2958.2008.06459.x 18990182

[84] Angelov A, Bergen P, Nadler F, Hornburg P, Lichev A, Ubelacker M, et al Novel Flp pilus biogenesis-dependent natural transformation. Front Microbiol. 2015;6:84 10.3389/fmicb.2015.00084 25713572 PMC4322843

[85] Pimentel ZT, Zhang Y. Evolution of the Natural Transformation Protein, ComEC, in Bacteria. Front Microbiol. 2018;9:2980 10.3389/fmicb.2018.02980 30627116 PMC6299819

[86] Claverys J-P, Martin B. Bacterial "competence" genes: signatures of active transformation, or only remnants? Trends in Microbiology. 2003;11(4):161–5. 12706993 10.1016/s0966-842x(03)00064-7

[87] Oliveira PH, Touchon M, Rocha EP. The interplay of restriction-modification systems with mobile genetic elements and their prokaryotic hosts. Nucleic Acids Res. 2014;42(16):10618–31. 10.1093/nar/gku734 25120263 PMC4176335

[88] Sinha S, Cameron AD, Redfield RJ. Sxy induces a CRP-S regulon in Escherichia coli. J Bacteriol. 2009;191(16):5180–95. 10.1128/JB.00476-09 19502395 PMC2725579

[89] Sinha S, Redfield RJ. Natural DNA uptake by Escherichia coli. PLoS ONE. 2012;7(4):e35620 10.1371/journal.pone.0035620 22532864 PMC3330819

[90] Ronish LA, Lillehoj E, Fields JK, Sundberg EJ, Piepenbrink KH. The structure of PilA from Acinetobacter baumannii AB5075 suggests a mechanism for functional specialization in Acinetobacter type IV pili. J Biol Chem. 2019;294(1):218–30. 10.1074/jbc.RA118.005814 30413536 PMC6322890

[91] Cehovin A, Simpson PJ, McDowell MA, Brown DR, Noschese R, Pallett M, et al Specific DNA recognition mediated by a type IV pilin. Proc Natl Acad Sci U S A. 2013;110(8):3065–70. 10.1073/pnas.1218832110 23386723 PMC3581936

[92] Cooper TF. Recombination speeds adaptation by reducing competition between beneficial mutations in populations of Escherichia coli. PLoS Biol. 2007;5(9):e225 10.1371/journal.pbio.0050225 17713986 PMC1950772

[93] Stahl FW, Murray NE. The evolution of gene clusters and genetic circularity in microorganisms. Genetics. 1966;53(3):569–76. 5331527 10.1093/genetics/53.3.569 PMC1211040

[94] Provvedi R, Dubnau D. ComEA is a DNA receptor for transformation of competent Bacillus subtilis. Mol Microbiol. 1999;31(1):271–80. 9987128 10.1046/j.1365-2958.1999.01170.x

[95] Burrows LL. Pseudomonas aeruginosa twitching motility: type IV pili in action. Annu Rev Microbiol. 2012;66:493–520. 10.1146/annurev-micro-092611-150055 22746331

[96] Craig L, Li J. Type IV pili: paradoxes in form and function. Curr Opin Struct Biol. 2008;18(2):267–77. 10.1016/j.sbi.2007.12.009 18249533 PMC2442734

[97] Makarova KS, Wolf YI, Forterre P, Prangishvili D, Krupovic M, Koonin EV. Dark matter in archaeal genomes: a rich source of novel mobile elements, defense systems and secretory complexes. Extremophiles. 2014;18(5):877–93. 10.1007/s00792-014-0672-7 25113822 PMC4158269

[98] Katoh K, Standley DM. MAFFT multiple sequence alignment software version 7: improvements in performance and usability. Mol Biol Evol. 2013;30(4):772–80. 10.1093/molbev/mst010 23329690 PMC3603318

[99] Criscuolo A, Gribaldo S. BMGE (Block Mapping and Gathering with Entropy): a new software for selection of phylogenetic informative regions from multiple sequence alignments. BMC Evol Biol. 2010;10:210 10.1186/1471-2148-10-210 20626897 PMC3017758

[100] Finn RD, Clements J, Eddy SR. HMMER web server: interactive sequence similarity searching. Nucleic Acids Res. 2011;39(Web Server issue):W29–37. 10.1093/nar/gkr367 21593126 PMC3125773

[101] Camacho C, Coulouris G, Avagyan V, Ma N, Papadopoulos J, Bealer K, et al BLAST+: architecture and applications. BMC Bioinformatics. 2009;10:421 10.1186/1471-2105-10-421 20003500 PMC2803857

[102] Miele V, Penel S, Duret L. Ultra-fast sequence clustering from similarity networks with SiLiX. BMC Bioinformatics. 2011;12:116 10.1186/1471-2105-12-116 21513511 PMC3095554

[103] Gouy M, Guindon S, Gascuel O. SeaView version 4: A multiplatform graphical user interface for sequence alignment and phylogenetic tree building. Mol Biol Evol. 2010;27(2):221–4. 10.1093/molbev/msp259 19854763

[104] Eddy SR. Accelerated Profile HMM Searches. PLoS Comput Biol. 2011;7(10):e1002195 10.1371/journal.pcbi.1002195 22039361 PMC3197634

[105] Dress AW, Flamm C, Fritzsch G, Grunewald S, Kruspe M, Prohaska SJ, et al Noisy: identification of problematic columns in multiple sequence alignments. Algorithms Mol Biol. 2008;3:7 10.1186/1748-7188-3-7 18577231 PMC2464588

[106] Hoang DT, Chernomor O, von Haeseler A, Minh BQ, Vinh LS. UFBoot2: Improving the Ultrafast Bootstrap Approximation. Mol Biol Evol. 2018;35(2):518–22. 10.1093/molbev/msx281 29077904 PMC5850222

[107] Kalyaanamoorthy S, Minh BQ, Wong TKF, von Haeseler A, Jermiin LS. ModelFinder: fast model selection for accurate phylogenetic estimates. Nat Methods. 2017;14(6):587–9. 10.1038/nmeth.4285 28481363 PMC5453245

[108] Lagesen K, Hallin P, Rodland EA, Staerfeldt HH, Rognes T, Ussery DW. RNAmmer: consistent and rapid annotation of ribosomal RNA genes. Nucleic Acids Res. 2007;35(9):3100–8. 10.1093/nar/gkm160 17452365 PMC1888812

[109] Capella-Gutierrez S, Silla-Martinez JM, Gabaldon T. trimAl: a tool for automated alignment trimming in large-scale phylogenetic analyses. Bioinformatics. 2009;25(15):1972–3. 10.1093/bioinformatics/btp348 19505945 PMC2712344

[110] Gascuel O. BIONJ: an improved version of the NJ algorithm based on a simple model of sequence data. Mol Biol Evol. 1997;14(7):685–95. 10.1093/oxfordjournals.molbev.a025808 9254330

[111] Shimodaira H. An approximately unbiased test of phylogenetic tree selection. Syst Biol. 2002;51(3):492–508. 10.1080/10635150290069913 12079646

[112] Szollosi GJ, Davin AA, Tannier E, Daubin V, Boussau B. Genome-scale phylogenetic analysis finds extensive gene transfer among fungi. Philos Trans R Soc Lond B Biol Sci. 2015;370(1678):20140335 10.1098/rstb.2014.0335 26323765 PMC4571573

