# Supplementary figures and images for: Diversification of the type IV filament superfamily into machines for adhesion, protein secretion, DNA uptake, and motility
Source: PLoS Biol. 2019 Jul 19;17(7):e3000390. doi: 10.1371/journal.pbio.3000390 (PMC6668835; doi:10.1371/journal.pbio.3000390)

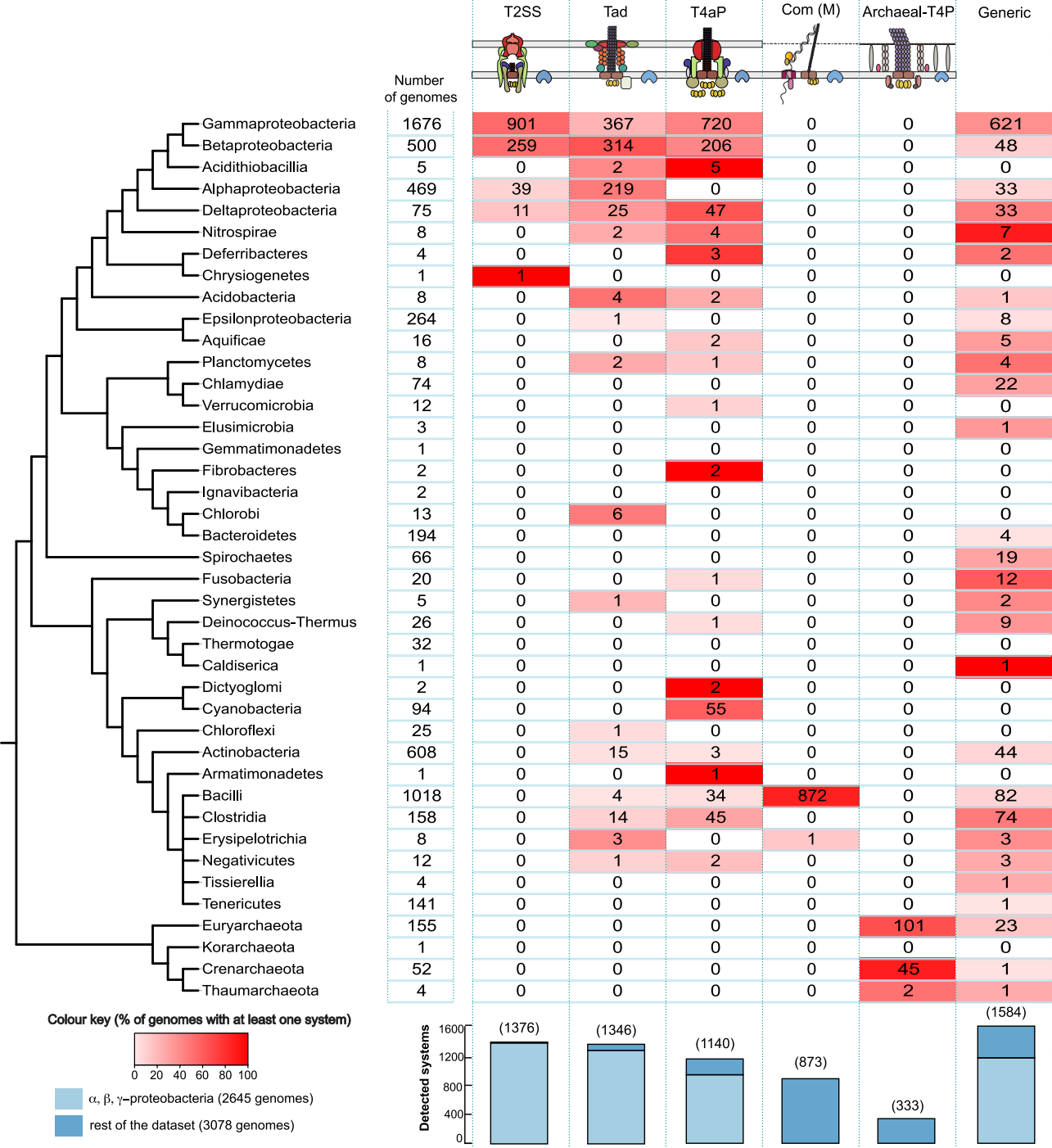

Supplement: S2 Fig — Cells indicate the number of genomes with at least one detected system. The cell’s colour gradient represents the proportion of genomes with at least one system in the clade. The bar plot shows the total number of detected systems. The bars are separated in two categories: Alpha-, Beta-, and Gamma-proteobacteria versus the other clades. The cladogram symbolises approximated relationships between the bacterial and archaeal taxa analysed in this study. (PDF) [file pbio.3000390.s002.pdf]

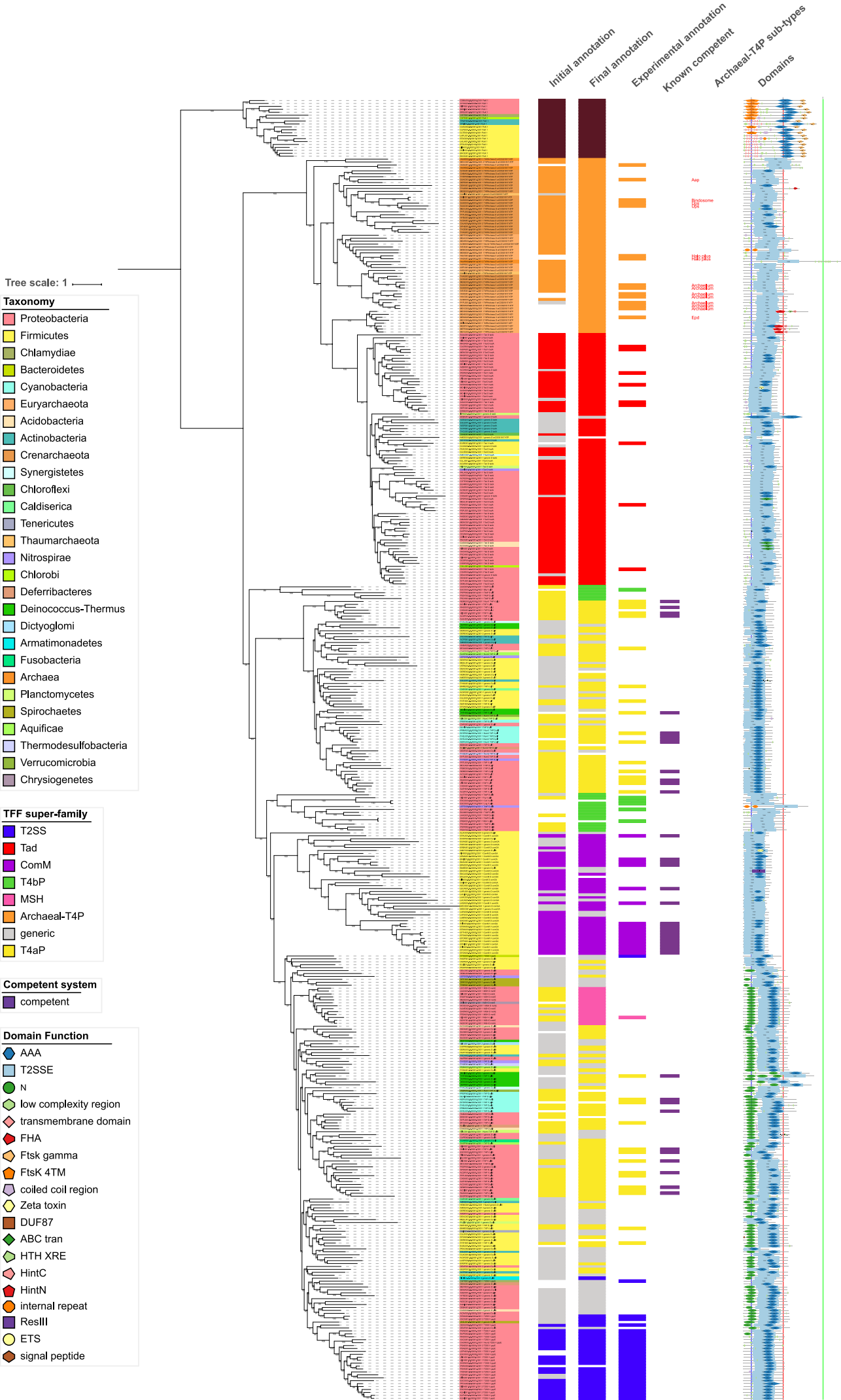

Supplement: S4 Fig — The colour of the label of the leaves indicates the taxonomic group of the species. The different coloured strips indicate the classification of the systems with the MacSyFinder annotation (with the initial model and with the final one) and the annotation of the systems in the literature. The systems known to be implicated in natural transformation are indicated in dark purple. Known subtypes of Archaeal-T4P are indicate by text in red. The annotation of the domains of the proteins using are also added. The tree was built using IQ-Tree, 10,000 replicates of UFBoot, model LG + R10. Halo pilus indicates two pili characterised in Halobacteria. Archaeal-T4P, type IV-related pili in Archaea; UFBoot, Ultrafast Bootstrap Approximation. (PDF) [file pbio.3000390.s004.pdf]

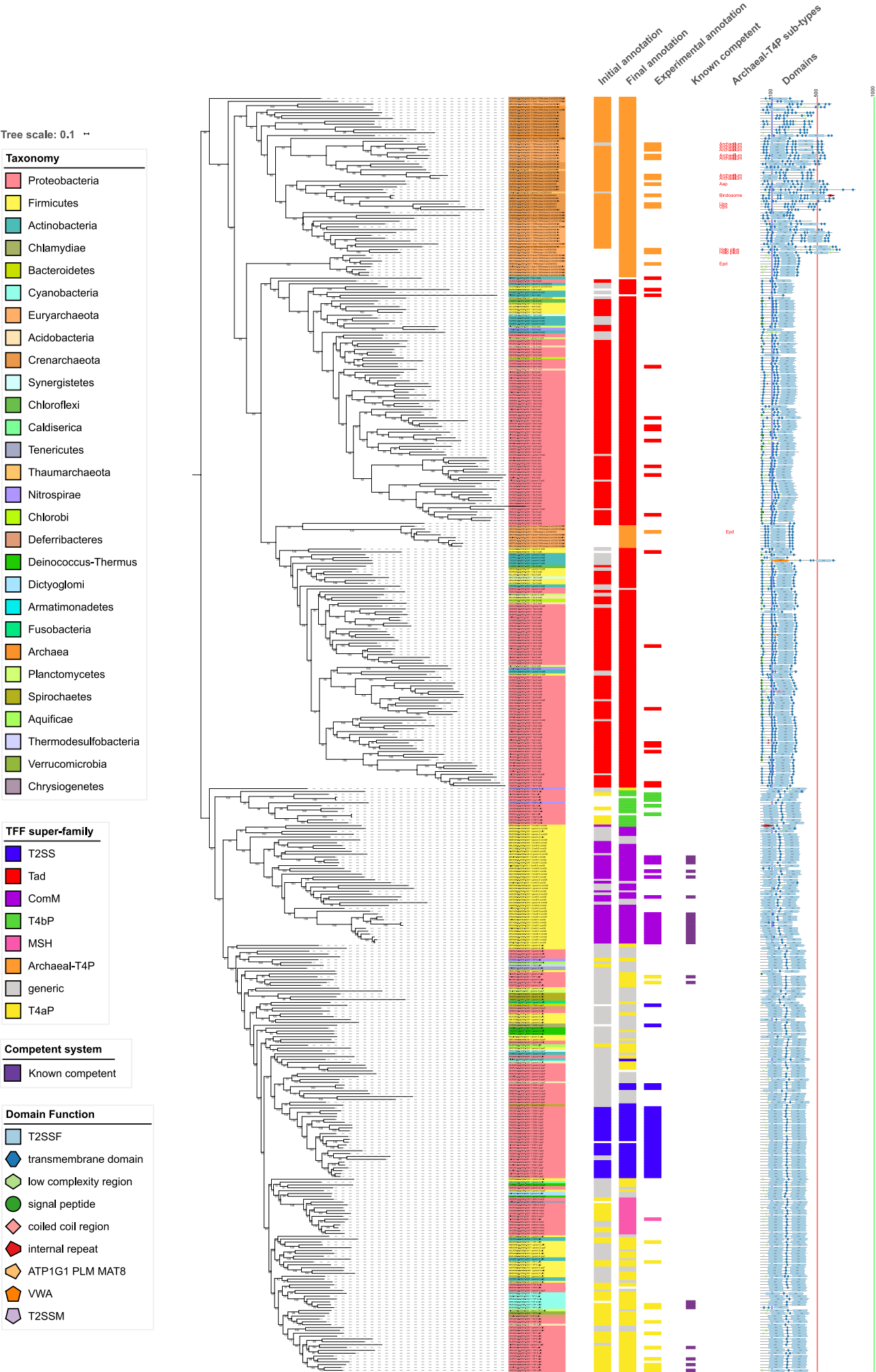

Supplement: S6 Fig — The colour of the label of the leaves indicates the taxonomic group of the species. The different coloured strips indicate the classification of the systems with the MacSyFinder annotation (with the initial model and with the final one) and the annotation of the systems in the literature. The systems known to be implicated in natural transformation are indicated in dark purple. Known subtypes of Archaeal-T4P are indicate by text in red. The annotation of the domains of the proteins using are also added. The tree was built using IQ-Tree, 10,000 replicates of UFBoot, model LG + F + R8. Halo pilus indicates two pili characterised in Halobacteria. Archaeal-T4P, type IV-related pili in Archaea; IM, integral membrane; UFBoot, Ultrafast Bootstrap Approximation. (PDF) [file pbio.3000390.s006.pdf]

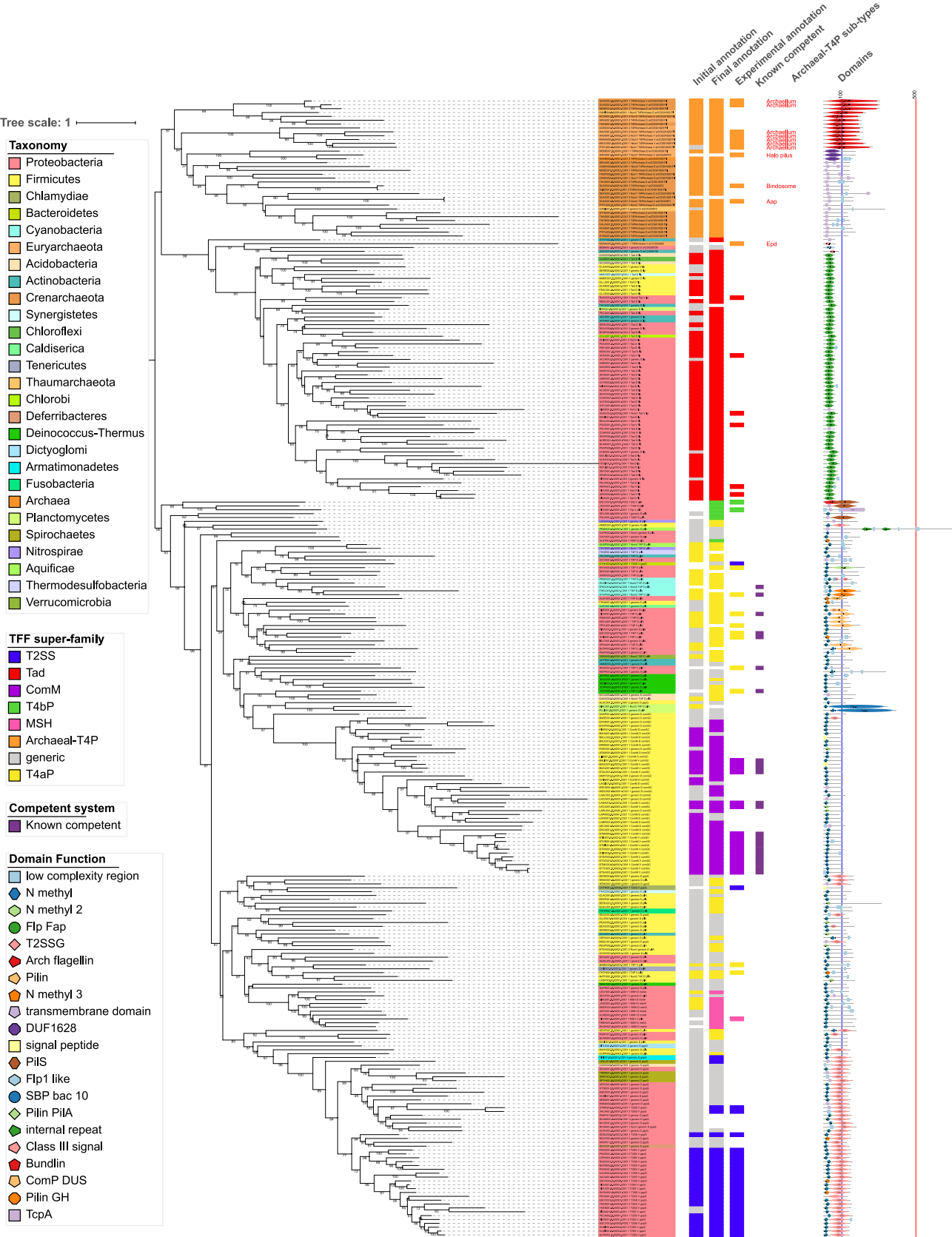

Supplement: S7 Fig — The colour of the label of the leaves indicates the taxonomic group of the species. The different coloured strips indicate the classification of the systems with the MacSyFinder annotation (with the initial model and with the final one) and the annotation of the systems in the literature. The systems known to be implicated in natural transformation are indicated in dark purple. Known subtypes of Archaeal-T4P are indicate by text in red. The annotation of the domains of the proteins using are also added. The tree was built using IQ-Tree, 10,000 replicates of UFBoot, model LG + F + R7. Halo pilus indicates two pili characterised in Halobacteria. Archaeal-T4P, type IV-related pili in Archaea; UFBoot, Ultrafast Bootstrap Approximation. (PDF) [file pbio.3000390.s007.pdf]

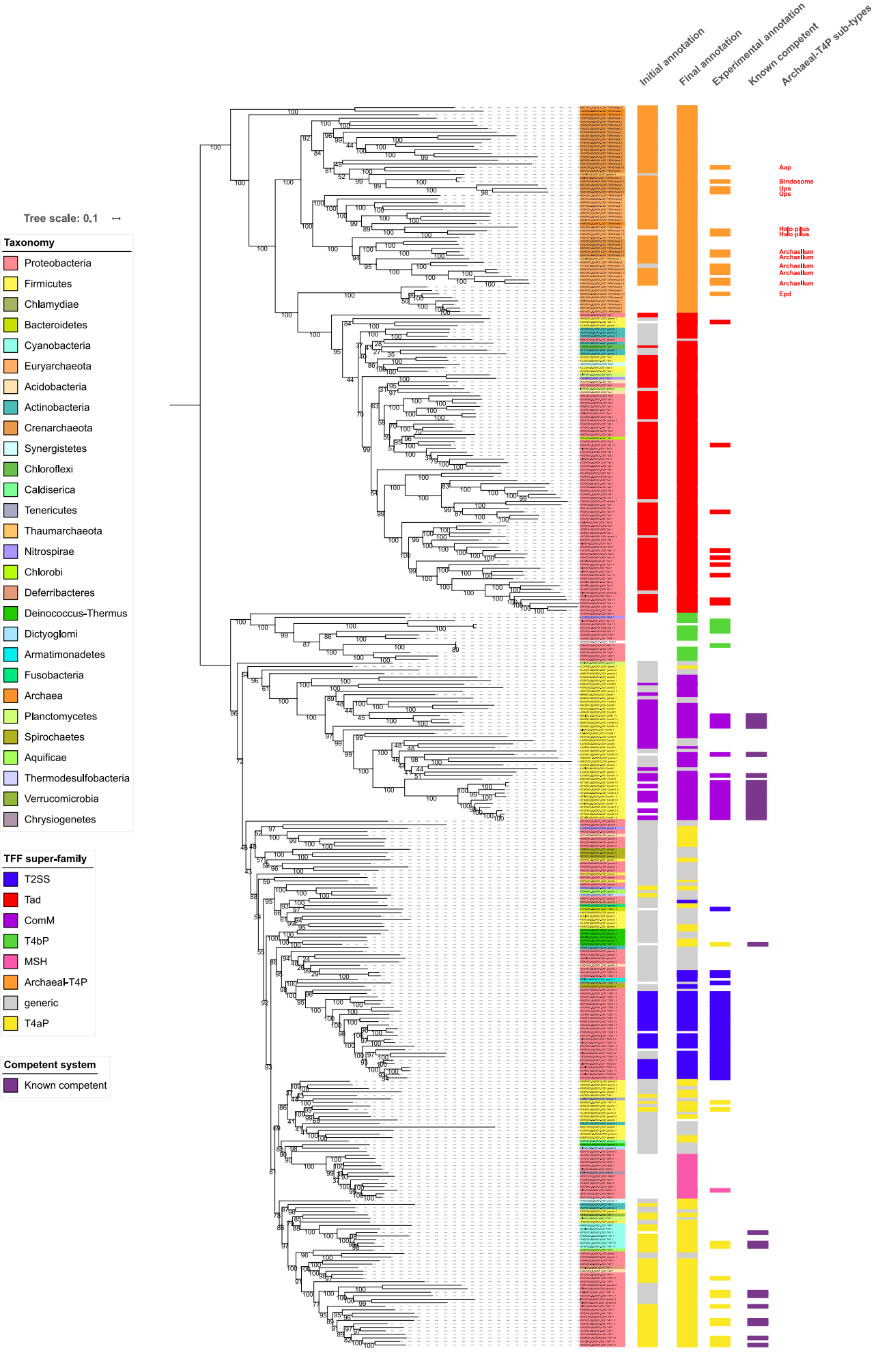

Supplement: S10 Fig — The tree was built with the concatenate of the IM platform (using TadB) and the AAA+ ATPase (using PilB). The colour of the label of the leaves indicates the taxonomic group of the species. The different coloured strips indicate the classification of the systems with the MacSyFinder annotation (with the initial model and with the final one) and the annotation of the systems in the literature. The systems known to be implicated in natural transformation are indicated in dark purple. Known subtypes of Archaeal-T4P are indicate by text in red. The tree was built using IQ-Tree, 10,000 replicates of UFBoot, with a partition model. Halo pilus indicates two pili characterised in Halobacteria. Archaeal-T4P, type IV-related pili in Archaea; Tad, tight adherence; TFF, type IV filament; UF, Ultrafast Bootstrap Approximation. (PDF) [file pbio.3000390.s010.pdf]

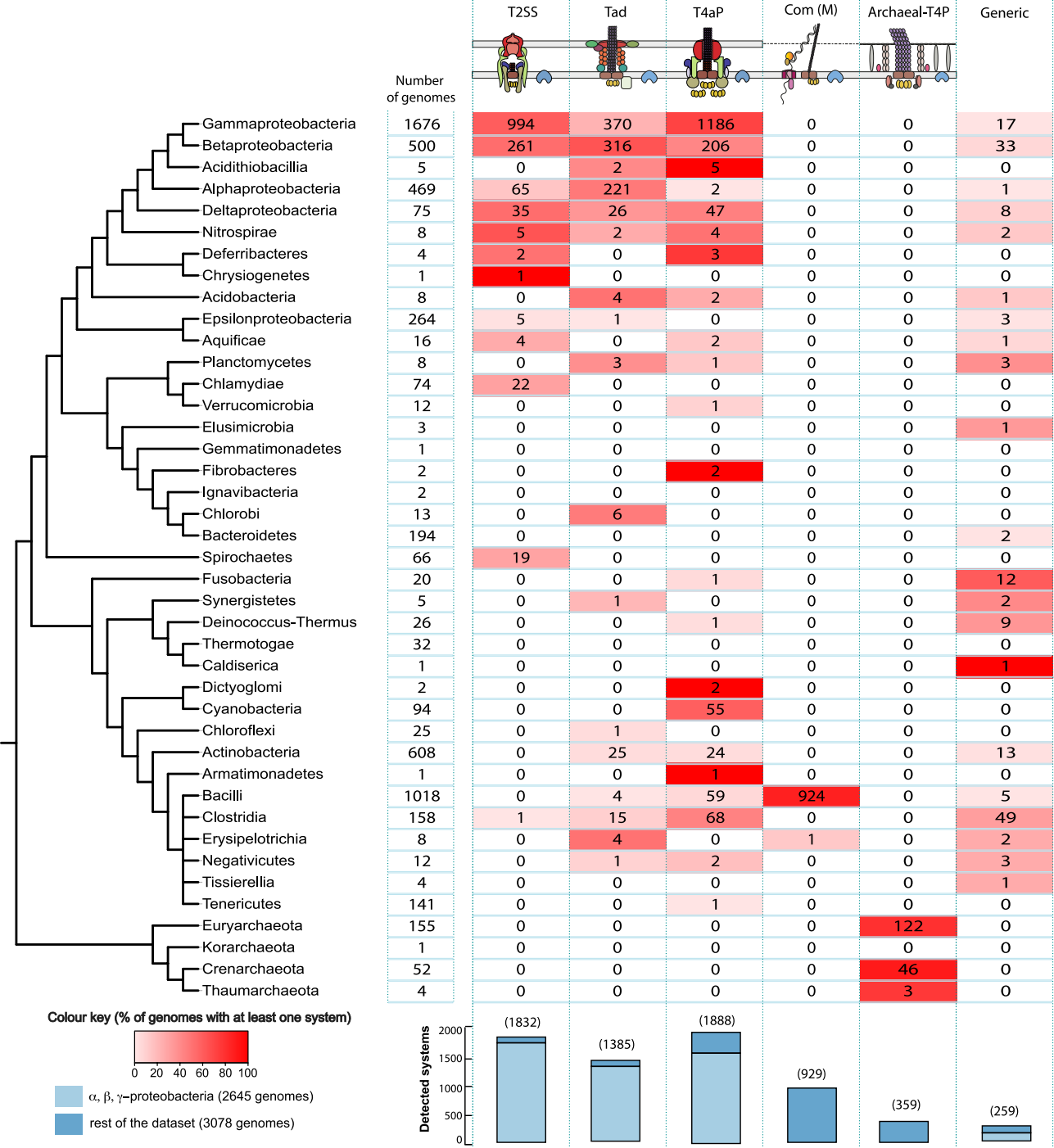

Supplement: S11 Fig — Cells indicate the number of genomes with at least one detected system. The cell’s colour gradient represents the proportion of genomes with at least one system in the clade. The bar plot shows the total number of detected systems. The bars are separated into two categories: Alpha-, Beta-, and Gamma-proteobacteria versus the other clades. The cladogram symbolises approximated relationships between the bacterial and archaeal taxa analysed in this study. (PDF) [file pbio.3000390.s011.pdf]

Node colour :

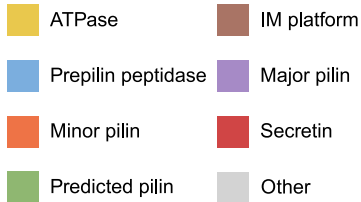

Edge width :

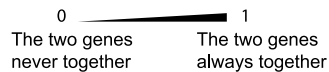

Edge colour :

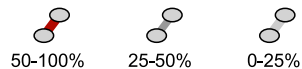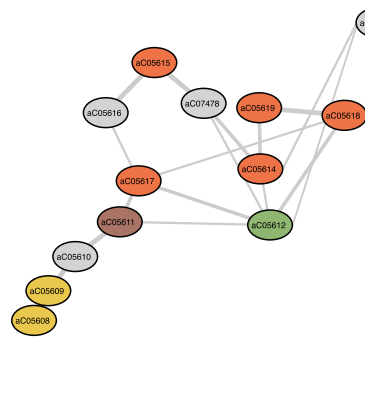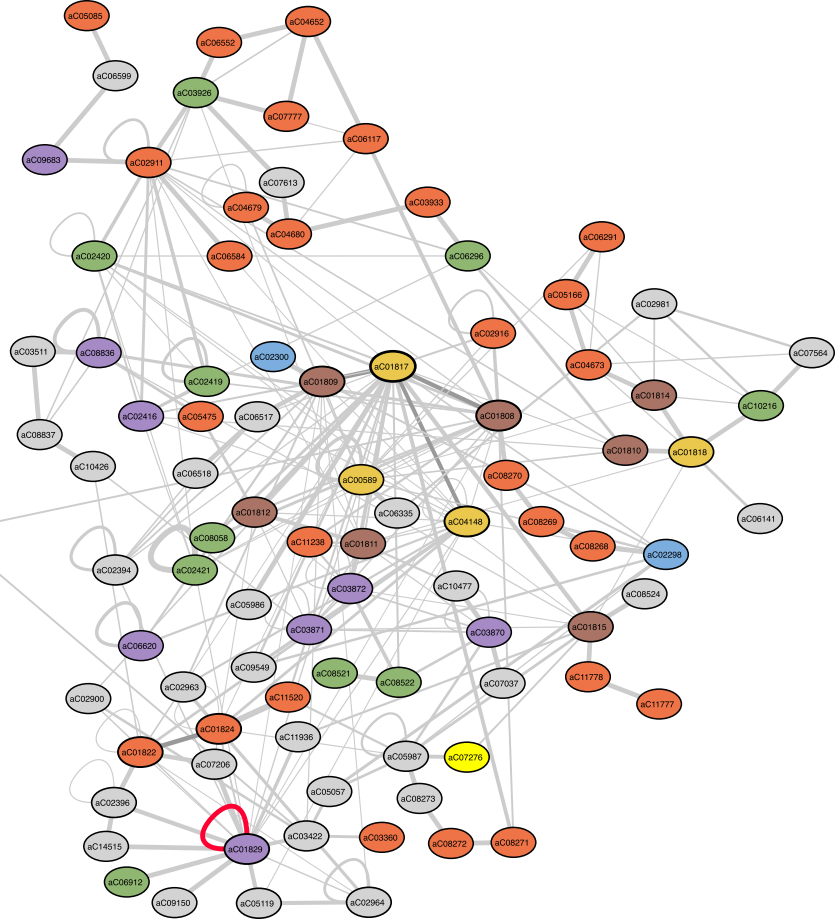

Supplement: S12 Fig — The edge width represents the number of times the two genes are contiguous divided by the number of times the rarest gene is present in the system. The colour of the edge represents the number of times the two genes are contiguous in the system divided by the number of systems. Archaeal-T4P, type IV-related pili in Archaea. (PDF) [file pbio.3000390.s012.pdf]

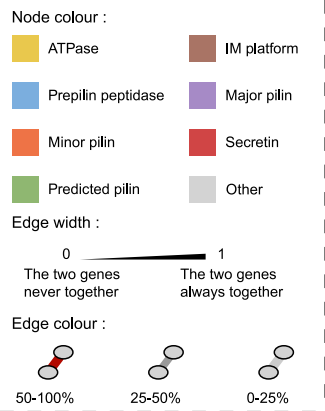

Archaeallum : aC01809 - aC01817 - aC04148 - aC01822 - aC01824

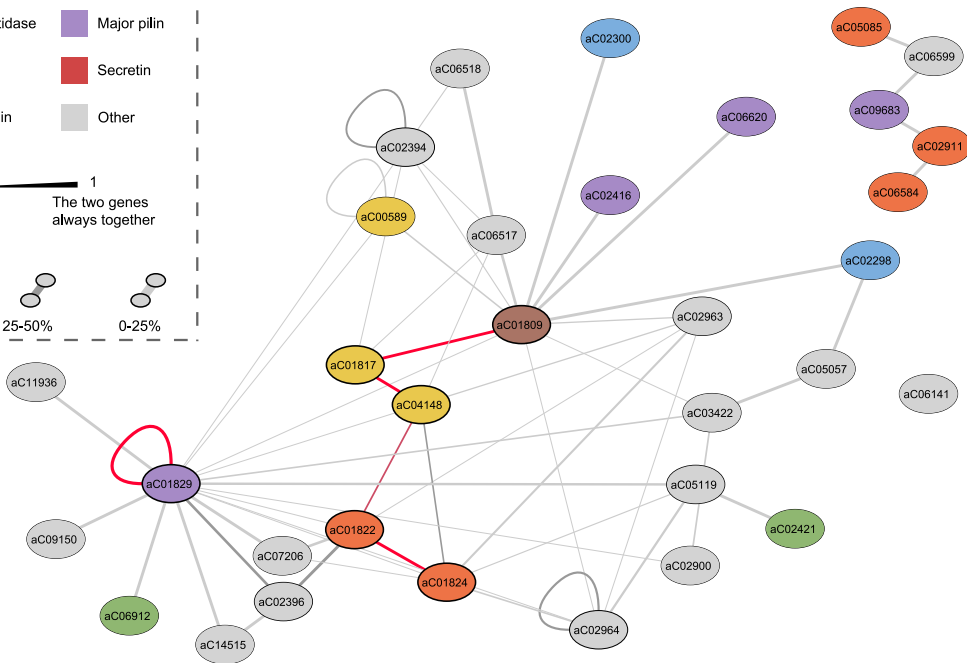

Supplement: S13 Fig — The edge width represents the number of times the two genes are contiguous divided by the number of times the rarest gene is present in the system. The colour of the edge represents the number of times the two genes are contiguous in the system divided by the number of systems. (PDF) [file pbio.3000390.s013.pdf]
