# Supplementary material for: Diversification of the type IV filament superfamily into machines for adhesion, protein secretion, DNA uptake, and motility
Source: PLoS Biol. 2019 Jul 19;17(7):e3000390. doi: 10.1371/journal.pbio.3000390 (PMC6668835; doi:10.1371/journal.pbio.3000390)
Supplement: S5 Fig — The colour of the label of the leaves indicates the taxonomic group of the species. The different coloured strips indicate the classification of the systems with the MacSyFinder annotation (with the initial model and with the final one) and the annotation of the systems in the literature. The systems known to be implicated in natural transformation are indicated in dark purple. Known subtypes of Archaeal-T4P are indicate by text in red. The annotation of the domains of the proteins using are also added. The tree was built using IQ-Tree, 10,000 replicates of UFBoot, model LG + R9. Halo pilus indicates two pili characterised in Halobacteria. Archaeal-T4P, type IV-related pili in Archaea; UFBoot, Ultrafast Bootstrap Approximation. (PDF) [file pbio.3000390.s005.pdf]

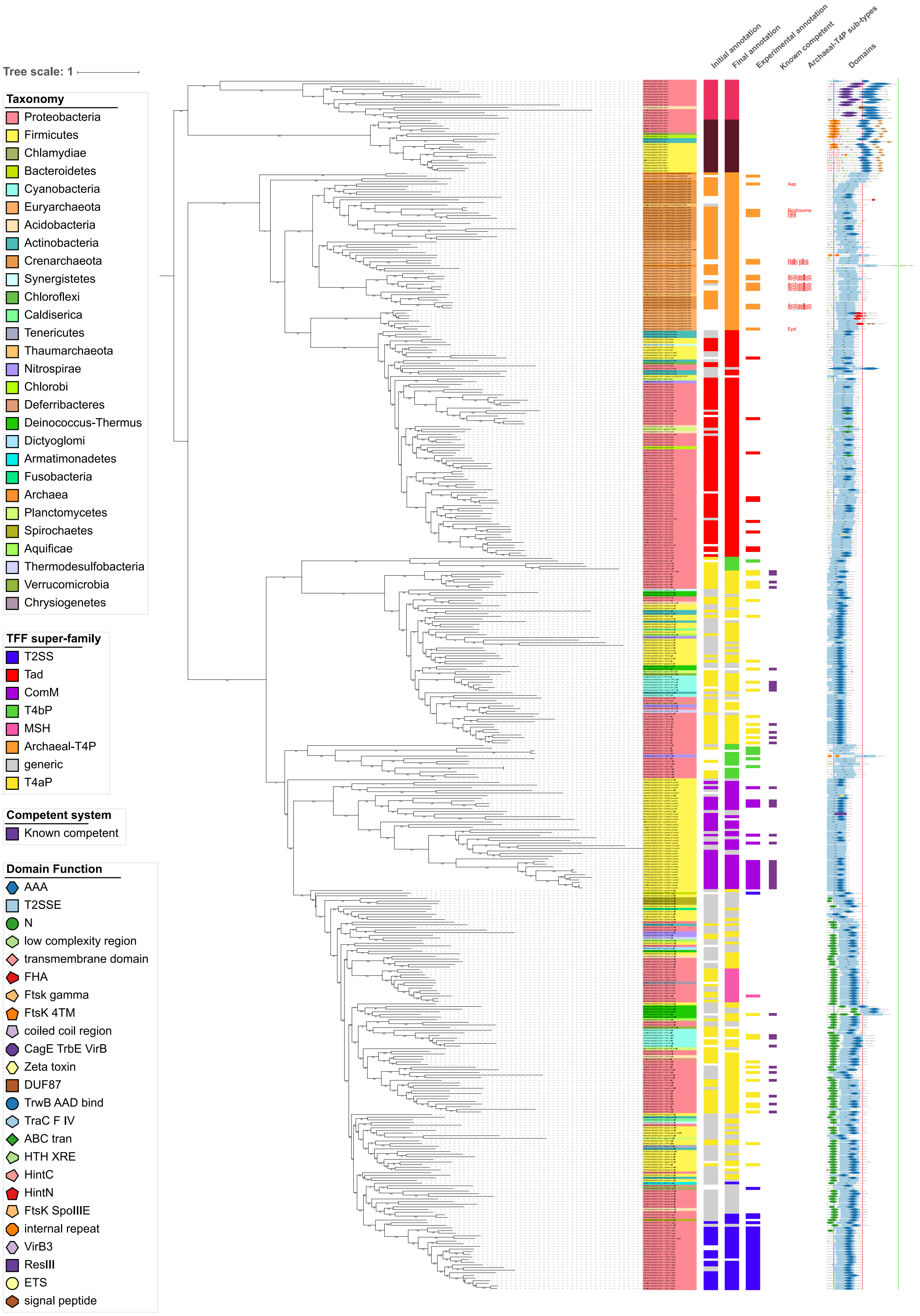
Tree scale: 1
Initial annotation
Final annotation
Experimental annotation
Known competent
Archaeal-T4P sub-types
Domains
Aap
Bindosome
Halo pilus
Archaellum
Epd
Taxonomy
Proteobacteria
Firmicutes
Chlamydiae
Bacteroidetes
Cyanobacteria
Euryarchaeota
Acidobacteria
Actinobacteria
Crenarchaeota
Synergistetes
Chloroflexi
Caldiserica
Tenericutes
Thaumarchaeota
Nitrospirae
Chlorobi
Deferribacteres
Deinococcus-Thermus
Dictyoglomi
Armatimonadetes
Fusobacteria
Archaea
Planctomycetes
Spirochaetes
Aquificae
Thermodesulfobacteria
Verrucomicrobia
Chrysiogenetes
TFF super-family
T2SS
Tad
ComM
T4bP
MSH
Archaeal-T4P
generic
T4aP
Competent system
Known competent
Domain Function
AAA
T2SSE
N
low complexity region
transmembrane domain
FHA
Ftsk gamma
FtsK 4TM
coiled coil region
CagE TrbE VirB
Zeta toxin
DUF87
TrwB AAD bind
TraC F IV
ABC tran
HTH XRE
HintC
HintN
FtsK SpoIIIE
internal repeat
VirB3
ResIII
ETS
signal peptide
