# Supplementary material for: Diversification of the type IV filament superfamily into machines for adhesion, protein secretion, DNA uptake, and motility
Source: PLoS Biol. 2019 Jul 19;17(7):e3000390. doi: 10.1371/journal.pbio.3000390 (PMC6668835; doi:10.1371/journal.pbio.3000390)
Supplement: S8 Fig — The colour of the label of the leaves indicates the taxonomic group of the species. The different coloured strips indicate the classification of the systems with the MacSyFinder annotation (with the initial model and with the final one) and the annotation of the systems in the literature. The systems known to be implicated in natural transformation are indicated in dark purple. Known subtypes of Archaeal-T4P are indicate by text in red. The annotation of the domains of the proteins used are also added. The tree was built using IQ-Tree, 10,000 replicates of UFBoot, model VT + F + R6. Archaeal-T4P, type IV-related pili in Archaea; UFBoot, Ultrafast Bootstrap Approximation. (PDF) [file pbio.3000390.s008.pdf]

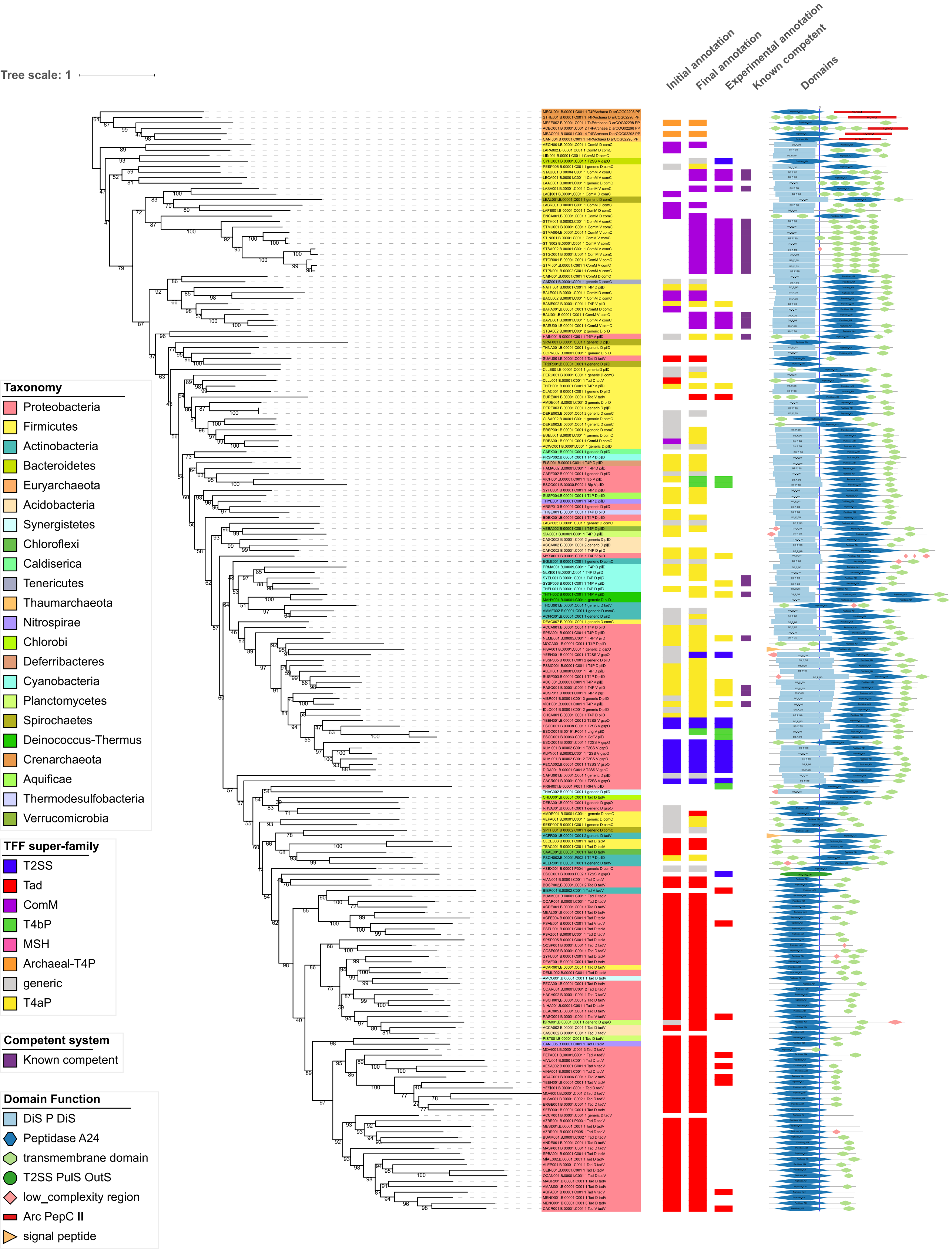
Tree scale: 1
Initial annotation
Final annotation
Experimental annotation
Known competent
Domains
Taxonomy
Proteobacteria
Firmicutes
Actinobacteria
Bacteroidetes
Euryarchaeota
Acidobacteria
Synergistetes
Chloroflexi
Caldiserica
Tenericutes
Thaumarchaeota
Nitrospirae
Chlorobi
Deferribacteres
Cyanobacteria
Planctomycetes
Spirochaetes
Deinococcus-Thermus
Crenarchaeota
Aquificae
Thermodesulfobacteria
Verrucomicrobia
TFF super-family
T2SS
Tad
ComM
T4bP
MSH
Archaeal-T4P
generic
T4aP
Competent system
Known competent
Domain Function
DiS P DiS
Peptidase A24
transmembrane domain
T2SS PulS OutS
low_complexity region
Arc PepC II
signal peptide
