# Supplementary material for: Diversification of the type IV filament superfamily into machines for adhesion, protein secretion, DNA uptake, and motility
Source: PLoS Biol. 2019 Jul 19;17(7):e3000390. doi: 10.1371/journal.pbio.3000390 (PMC6668835; doi:10.1371/journal.pbio.3000390)
Supplement: S9 Fig — The colour of the label of the leaves indicates the taxonomic group of the species. The different coloured strips indicate the classification of the systems with the MacSyFinder annotation (with the initial model and with the final one) and the annotation of the systems in the literature. The systems known to be implicated in natural transformation are indicated in dark purple. Known subtypes of Archaeal-T4P are indicate by text in red. The annotation of the domains of the proteins used are also added. The tree was built using IQ-Tree, 10,000 replicates of UFBoot, model LG + F + R8. Archaeal-T4P, type IV-related pili in Archaea; UFBoot, Ultrafast Bootstrap Approximation. (PDF) [file pbio.3000390.s009.pdf]

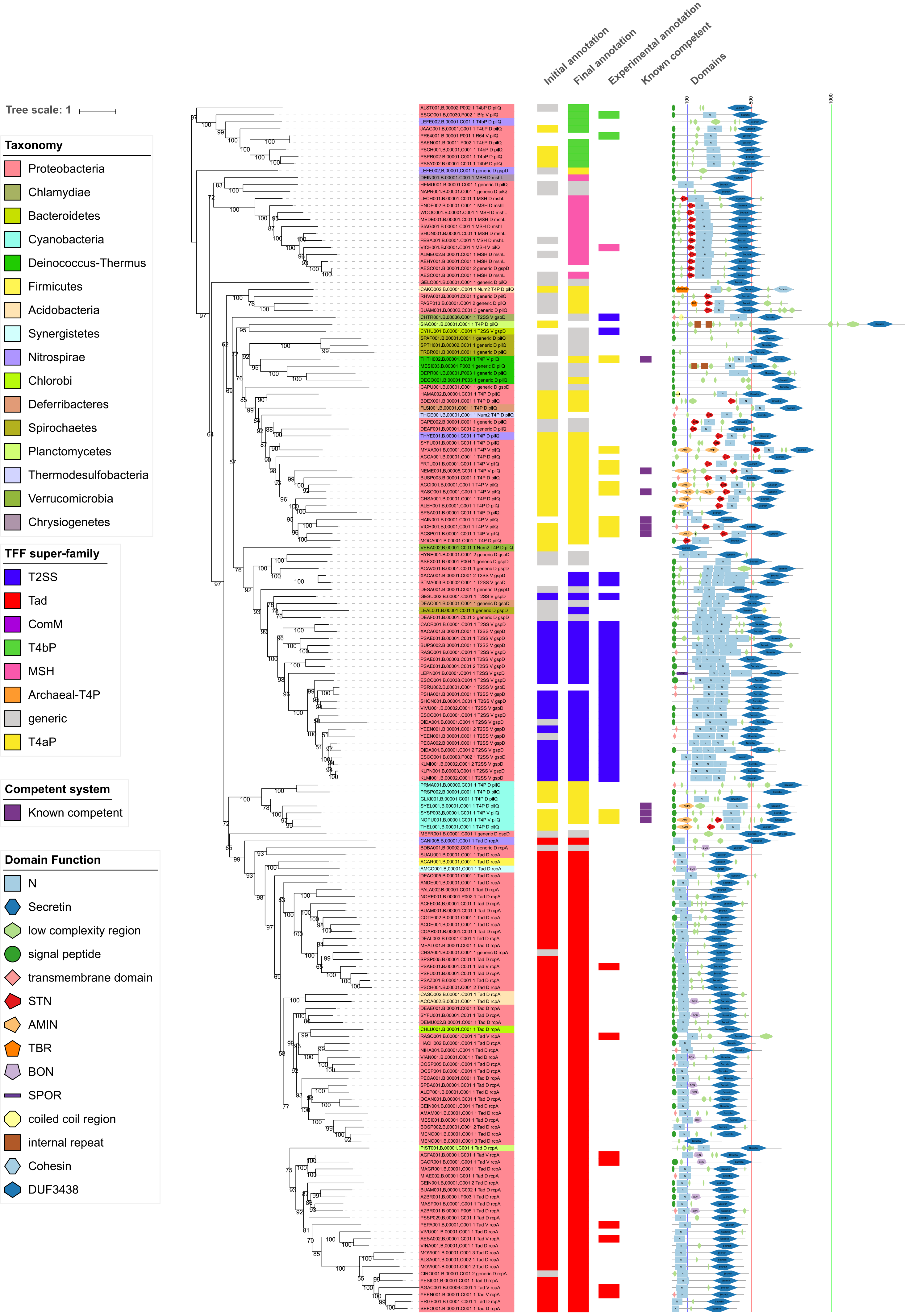

Initial annotation
Final annotation
Experimental annotation
Known competent
Domains
Tree scale: 1
Taxonomy
Proteobacteria
Chlamydiae
Bacteroidetes
Cyanobacteria
Deinococcus-Thermus
Firmicutes
Acidobacteria
Synergistetes
Nitrospirae
Chlorobi
Deferribacteres
Spirochaetes
Planctomycetes
Thermodesulfobacteria
Verrucomicrobia
Chrysiogenetes
TFF super-family
T2SS
Tad
ComM
T4bP
MSH
Archaeal-T4P
generic
T4aP
Competent system
Known competent
Domain Function
N
Secretin
low complexity region
signal peptide
transmembrane domain
STN
AMIN
TBR
BON
SPOR
coiled coil region
internal repeat
Cohesin
DUF3438
