# Supplementary material for: Diversification of the type IV filament superfamily into machines for adhesion, protein secretion, DNA uptake, and motility
Source: PLoS Biol. 2019 Jul 19;17(7):e3000390. doi: 10.1371/journal.pbio.3000390 (PMC6668835; doi:10.1371/journal.pbio.3000390)
Supplement: S14 Fig — The colour of the leaves represents the phyla of the Bacteria. The tree was built using IQ-Tree, 1,000 replicates of UFBoot, model SYM + R10. UFBoot, Ultrafast Bootstrap Approximation. (PDF) [file pbio.3000390.s014.pdf]

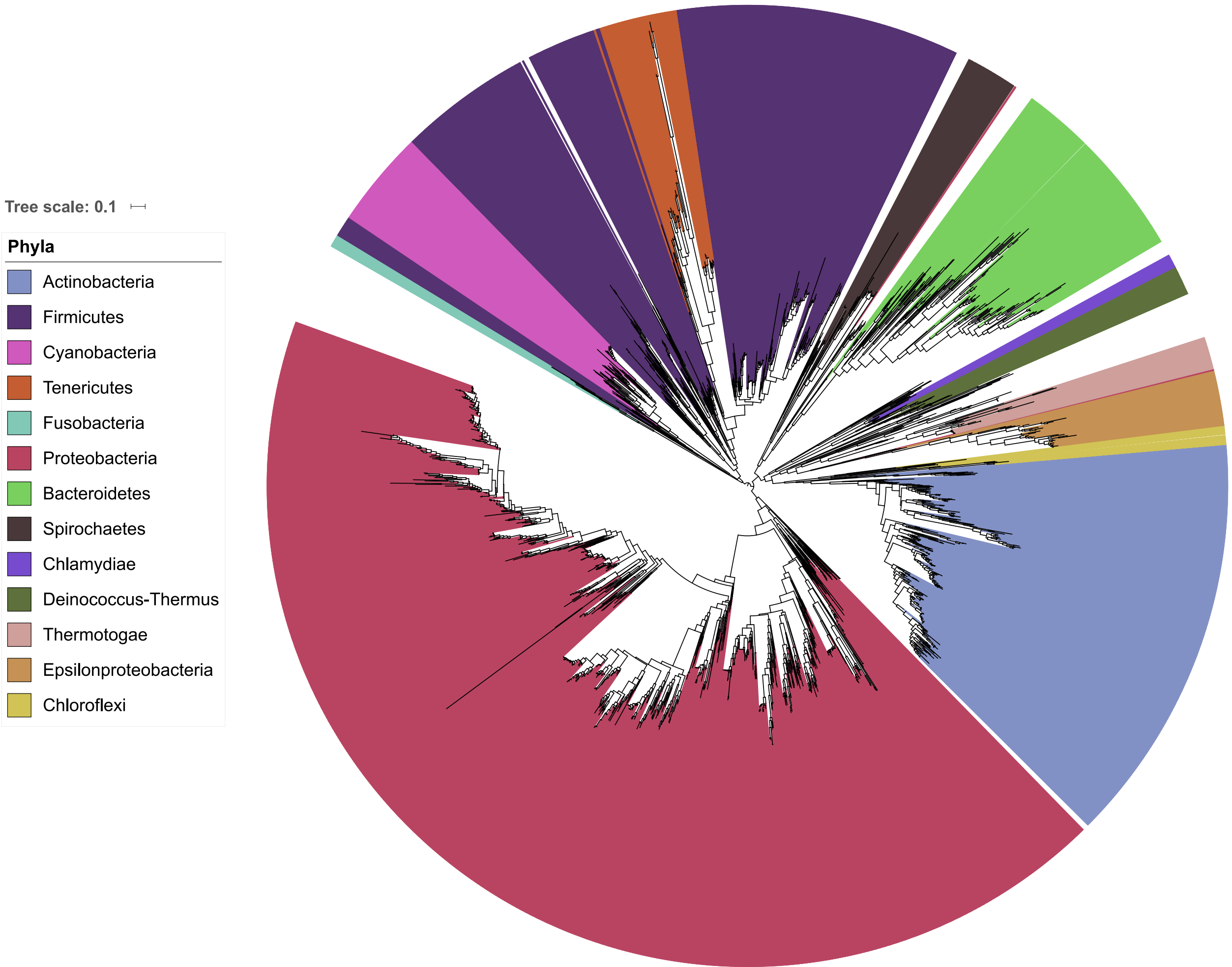

Tree scale: 0.1
Phyla
Actinobacteria
Firmicutes
Cyanobacteria
Tenericutes
Fusobacteria
Proteobacteria
Bacteroidetes
Spirochaetes
Chlamydiae
Deinococcus-Thermus
Thermotogae
Epsilonproteobacteria
Chloroflexi
