# Supplementary material for: Diversification of the type IV filament superfamily into machines for adhesion, protein secretion, DNA uptake, and motility
Source: PLoS Biol. 2019 Jul 19;17(7):e3000390. doi: 10.1371/journal.pbio.3000390 (PMC6668835; doi:10.1371/journal.pbio.3000390)
Supplement: S15 Fig — (PDF) [file pbio.3000390.s015.pdf]

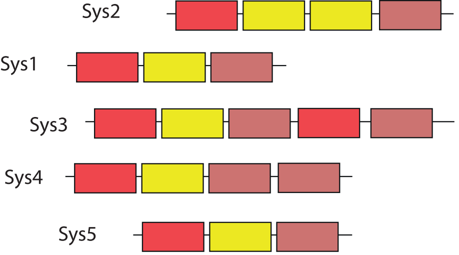

Step 1 : On the systems with at least ATPase and IM-platform protein

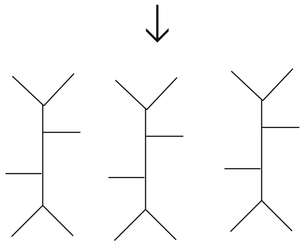

Step 2 : Inferring ML tree for each "core protein" family of the systems

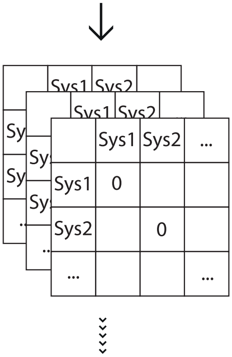

Step 3a : Extracting patristic distances for all the trees

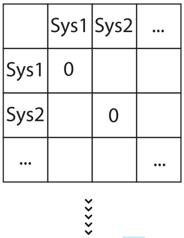

Step 3b : Inferring a bioNJ tree with the patristic distance

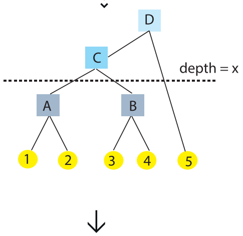

Step 3c : Cutting the tree at a fixed depth

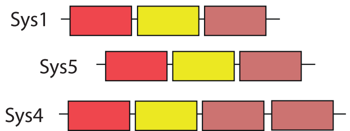

Step 4 : Selecting one system for each cluster
