# Supplementary material for: Diversification of the type IV filament superfamily into machines for adhesion, protein secretion, DNA uptake, and motility
Source: PLoS Biol. 2019 Jul 19;17(7):e3000390. doi: 10.1371/journal.pbio.3000390 (PMC6668835; doi:10.1371/journal.pbio.3000390)
Supplement: S16 Fig — (PDF) [file pbio.3000390.s016.pdf]

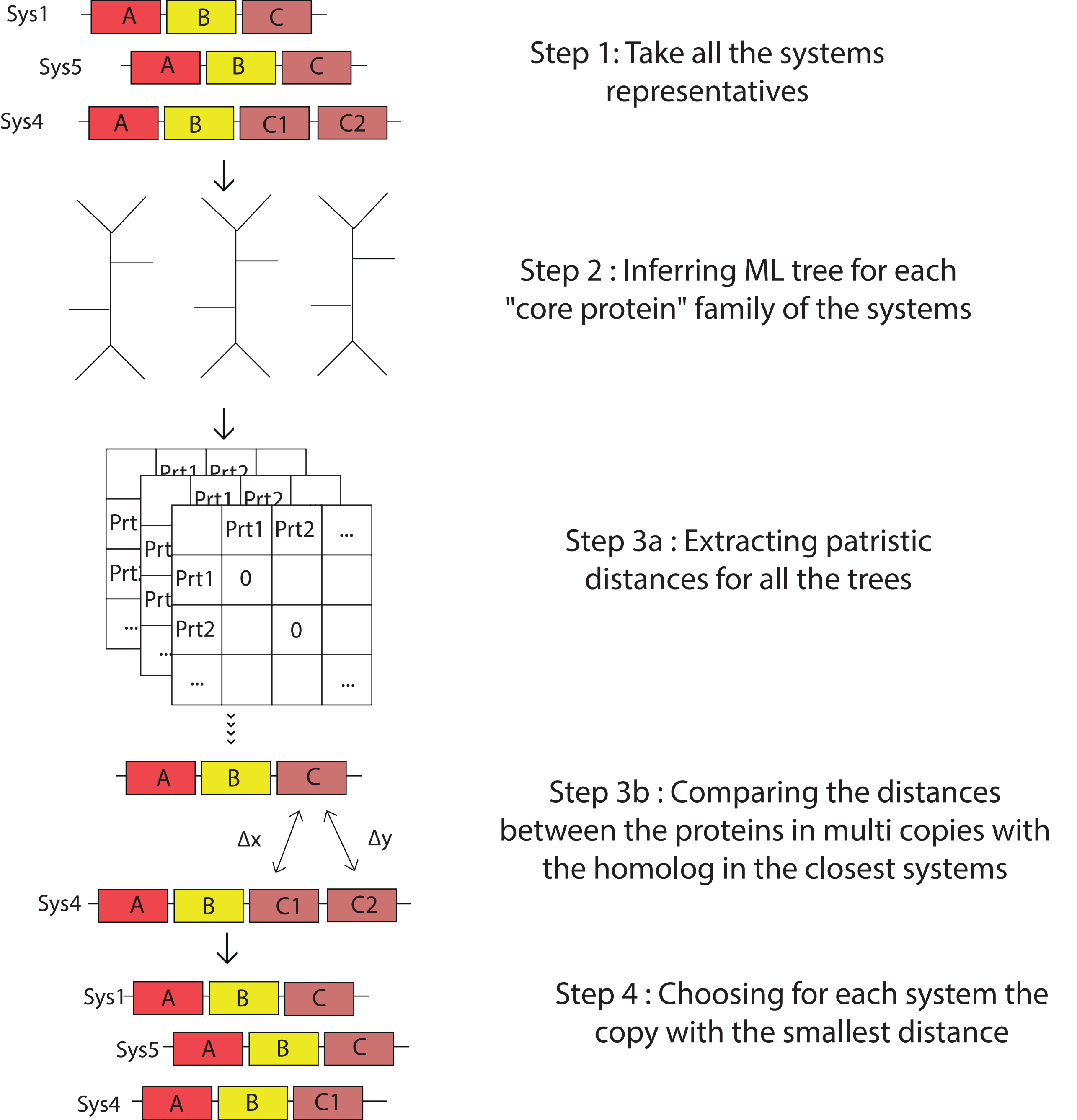
Sys1
A
B
C
Sys5
A
B
C
Sys4
A
B
C1
C2
Step 1: Take all the systems representatives
Step 2 : Inferring ML tree for each "core protein" family of the systems
Prt1
Prt2
...
0
0
Step 3a : Extracting patristic distances for all the trees
A
B
C
Δx
Δy
Sys4
A
B
C1
C2
Step 3b : Comparing the distances between the proteins in multi copies with the homolog in the closest systems
Sys1
A
B
C
Sys5
A
B
C
Sys4
A
B
C1
Step 4 : Choosing for each system the copy with the smallest distance
