# Supplementary material for: Diversification of the type IV filament superfamily into machines for adhesion, protein secretion, DNA uptake, and motility
Source: PLoS Biol. 2019 Jul 19;17(7):e3000390. doi: 10.1371/journal.pbio.3000390 (PMC6668835; doi:10.1371/journal.pbio.3000390)
Supplement: S8 Table — TFF, type IV filament. (PDF) [file pbio.3000390.s024.pdf]

S8 Table. Global scenario of TFF evolution: summary of the evolutionary events presented in Fig 7

Alternatives were only suggested when not invoking too complicated events to explain all observations, when compared to the proposed scenario.

Step	Proposed scenario	Justification	Alternatives
1	Presence of the secretin in the ancestral TFF of Bacteria	T4bP have secretins and are the sister lineage to TFF with (T4aP in diderms and T2SS) and without secretins (ComM). The phylogeny of the secretin (Fig. S9) recapitulates that of the bacterial TFF. It is more parsimonious to infer the presence of the secretin in the ancestor of bacterial TFF.	Multiple independent gains of secretins in T4aP and T4bP. This is not parsimonious since it does not explain easily how the secretin tree follows the history of TFF.
2	Duplication of the PilB/PilT ATPase in the ancestral TFF of Bacteria	Most parsimonious explanation for their presence in both T4bP and T4aP. The phylogeny of the ATPase has PilT from T4aP and T4bP on one side, and PilB from T4bP, ComM, T4aP, MSH and T2SS on the other side suggesting ancestral duplication (subtrees for paralogs are congruent).	No simple alternative explanation. Some trees provide complex scenarios, where there were several duplications of the ancestor of PilB/PilT. While these are less compatible with the phylogeny, they would explain lack of PilT in ComM (but not lack in T2SS or MSH, since these are derived from T4aP).
3	Losses of the PilT ATPase in the ancestors of ComM, T2SS and MSH.	See Step 6.	See Step 2.
4	Loss of the secretin in ComM's ancestor	See Step 5.	See Step 1.
5	Acquisition of the assembly proteins PilMNO in the ancestor of T4aP, T2SS and MSH	These proteins have known homologs only in T4aP, MSH and T2SS.	No simple alternative explanation.
6	Acquisition of MshN in the ancestral MSH	*MshN* is specific to MSH and is largely represented in MSH loci, therefore it is most parsimonious to infer its acquisition in MSH's ancestor.	No simple alternative explanation.
7	Duplication of the PilT/PilU ATPase in T4aP	PilU is present in only a subset of T4aP. This is consistent with the phylogeny of ATPase, as PilU group together within PilT.	No simple alternative explanation

8	Fission of the IM platform gene in the ancestor of Epd/Tad	The IM platform has 2 domains T2SSF in the other archaeal and bacterial homologs, but 2 proteins with one T2SSF domain in Epd and Tad. They align resp. to C-ter (TadB) and N-Ter (TadC) of the 2-domains proteins. The phylogenies of TadB and TadC are similar and so is that of their concatenate.	Duplication of the IM platform in the ancestor of Epd followed by the loss of a domain in each paralog (one in N-ter, the other in C-ter). It is less parsimonious as it invokes 1 duplication and 2 losses, versus 1 fission for the proposed scenario.
9	Transfer of Tad's ancestor from Archaea to Bacteria	Tad robustly branches within archaeal systems, together with the Epd pilus in both individual and concatenate trees. Tad shares several derived features with Epd and other archaeal systems: the fission of IM platform (see Step 1). Presence of homologs of the protein family consisting of TadZ/ arCOG00589/ arCOG05608 - that has no homologs in other Bacterial TFF. Similar genetic organization (Epd).	No parsimonious alternative explains all the observed patterns.
10	Acquisition of the secretin in Tad's ancestor	Diderms branch first for Tad in 8 out of 10 of our concatenate trees, suggesting that the secretin was acquired early in Tad's history. Secretins in Tad of Firmicutes branch with the other Tad secretins.	Tad acquired initially by a monoderm that upon transfer to a diderm acquired a secretin. That would be in contradiction with most of the concatenate trees, and is not more parsimonious.
11	Acquisition of TadD in the ancestral Tad	*TadD* is specific to the Tad and is largely represented in Tad loci, therefore it is most parsimonious to infer its acquisition in Tad’s ancestor.	No simple alternative explanation.
12	Loss(es) of the secretin in Tad of monoderms	The secretin, and concatenate trees for Step 3 suggest multiple losses of the secretin in the Tad of monoderms (mostly Firmicutes and Actinobacteria).	See the alternative to Step 10.
